# A Meta-Analysis of Randomized Controlled Trials (RCTs) Investigating the Efficacy and Safety of Acupuncture in Treating Myocardial Ischemia/Reperfusion (I/R) Injury

**DOI:** 10.1155/crp/9970541

**Published:** 2025-06-24

**Authors:** Jian Xiong, Ying Wei, Xiaogang Huang, Jinqun Hu, Fayang Ling, Zhihao Shang, Wenchuan Qi, Qianhua Zheng, Dehua Li, Fanrong Liang

**Affiliations:** ^1^College of Acupuncture and Tuina, Chengdu University of Traditional Chinese Medicine, Chengdu, Sichuan, China; ^2^The First College of Clinical Medicine, Guangxi University of Traditional Chinese Medicine, Nanning, Guangxi, China; ^3^The First College of Clinical Medicine, Nanjing University of Traditional Chinese Medicine, Nanjing, Jiangsu, China; ^4^Acupuncture Department, The Affiliated Hospital of Chengdu University of Traditional Chinese Medicine, Chengdu, Sichuan, China

**Keywords:** acupuncture, cardiac surgery with cardiopulmonary bypass, meta-analysis, myocardial ischemia/reperfusion injury

## Abstract

**Objectives:** This study systematically reviewed and meta-analyzed randomized controlled trials (RCTs) evaluating the efficacy and safety of acupuncture in myocardial ischemia/reperfusion (I/R) injury.

**Methods:** A comprehensive literature search was conducted in PubMed, Cochrane Library, Web of Science, Chinese National Knowledge Infrastructure, China Science and Technology Journal Database, and Wanfang from database inception to November 3, 2024. Eligible RCTs assessing acupuncture for myocardial I/R injury were included. Statistical analyses were performed using Review Manager 5.3 and Stata 16.

**Results:** A total of 26 RCTs of moderate methodological quality were included. Acupuncture significantly reduced myocardial enzyme levels compared to controls. Inflammatory markers (hs-CRP, TNF-α, IL-6, IL-8, and IL-1) were suppressed, while anti-inflammatory and immunoregulatory factors (IL-10 and IL-2) increased. Oxidative stress parameters showed improvements, with reductions in MDA and SOD levels. Echocardiographic findings demonstrated enhanced cardiac function, reflected by increased LVEF and LVESV, along with reductions in LVFS, LVEDD, LVEDV, and LVESD. Additionally, acupuncture alleviated TCM chest pain symptoms, shortened ICU stays, lowered MACE incidence, and improved 6MWT and SAQ indicators. No adverse reactions were reported.

**Conclusion:** Acupuncture attenuates myocardial injury, inflammation, and oxidative stress while activating anti-inflammatory and immune responses, enhancing cardiac function, and mitigating ventricular remodeling. Furthermore, it alleviates chest pain, shortens ICU stays, reduces adverse cardiovascular events, and improves 6MWT and SAQ indicators.

## 1. Introduction

Myocardial ischemia-reperfusion (I/R) injury is a prevalent clinical cardiovascular disorder. Following a period of ischemic myocardial blood supply interruption, subsequent restoration of perfusion does not effectively repair the damaged tissue but instead exacerbates myocardial injury, inducing further lesions and necrosis [1]. The resulting damage spans various critical functions, including cardiac pump efficiency, electrophysiological stability, myocardial fiber ultrastructure, and energy metabolism, ultimately leading to irreversible cardiomyocyte apoptosis, enhanced necrosis, and infarct expansion [2, 3]. Myocardial I/R injury frequently precipitates complications such as cardiogenic shock, arrhythmias, and other adverse cardiovascular events, posing significant risks to patient survival and health [4]. It is commonly associated with procedures such as coronary angioplasty, thrombolytic therapy for myocardial infarction, and cardiac surgery [5]. The injury occurs in two distinct phases: initial coronary artery occlusion leads to a dramatic reduction in myocardial perfusion, inducing ischemia that triggers apoptosis and necrosis, ultimately resulting in myocardial cell death. In contrast, reperfusion, which involves the sudden influx of blood into the ischemic tissue, further exacerbates the condition by inducing oxidative stress, calcium (Ca^2+^) overload, and inflammation. These processes promote cardiomyocyte apoptosis and intensify damage to ischemic myocardial tissue, complicating the management of ischemic cardiomyopathy, worsening the patient's condition, and ultimately affecting the prognosis [6]. The global incidence of cardiovascular disease continues to rise, establishing it as the leading cause of death worldwide [7]. According to the World Health Organization (WHO), ischemic heart disease (IHD) remains the primary cause of mortality and disability globally. In 2019, IHD affected 197 million people, resulting in 9.14 million deaths, which accounted for 16% of global mortality [8]. Deaths from acute myocardial infarction have increased by 22.3% over the past decade, imposing a substantial health and economic burden on society [9]. Despite advancements in early vascular recanalization technologies, the secondary myocardial injury induced by reperfusion significantly impacts the prognosis of myocardial infarction patients. Clinical and laboratory studies [10, 11] have demonstrated that myocardial I/R injury heightens the risk of severe cardiovascular events, including heart failure, recurrent myocardial infarction, renal failure, in-stent restenosis, cerebrovascular accidents, and mortality. Targeting the molecular pathways associated with myocardial I/R injury offers a promising approach to reducing the incidence of such adverse outcomes.

Modern therapeutic approaches for myocardial I/R injury primarily encompass physical and pharmacological interventions, both of which have demonstrated varying degrees of efficacy in clinical and preclinical studies [3, 12–15]. However, physical therapies—including postischemic conditioning, hyperbaric oxygen therapy, hypothermia therapy, remote ischemic conditioning, and exercise training—face significant limitations related to ethical concerns, environmental constraints, feasibility of widespread application, and insufficient high-quality evidence from large-scale clinical trials. Pharmacological treatments remain largely confined to the preclinical stage, with many compounds exhibiting promising effects only in animal models. Moreover, the adverse effects associated with certain drugs substantially restrict their clinical utility. As a result, the development of a safe and effective treatment for myocardial I/R injury remains a significant challenge. Acupuncture, a fundamental therapeutic modality in traditional Chinese medicine (TCM), which involves the precise stimulation of acupoints using specialized instruments, combined with manipulation techniques intrinsic to TCM. In recent years, acupuncture has gained attention as a safe, cost-effective, and promising nonpharmacological intervention for cardiovascular diseases, including angina pectoris [16], hypertension [17], and arrhythmia [18]. Between 2000 and 2020, 2471 systematic reviews on acupuncture were published in the Science Network database, of which 235 (9.5%) focused on cardiovascular diseases [19]. Clinically, electroacupuncture at Neiguan (PC6), Lieque (LU7), and Yunmen (LU2) has shown to reduce serum cardiac troponin I (cTnI) levels, enhance muscle strength scores, and shorten intensive care unit (ICU) stays in adult patients undergoing cardiac valve replacement, thereby mitigating myocardial I/R injury [20]. Additionally, transcutaneous electrical acupoint stimulation at bilateral Neiguan (PC6) has been reported to significantly lower serum cTnI and C-reactive protein (CRP) levels in pediatric patients undergoing cardiac surgery, effectively reducing myocardial damage [21]. Despite these promising findings, no comprehensive evidence-based evaluation has been conducted to objectively assess the clinical efficacy and safety of acupuncture in the management of myocardial I/R injury. To address this gap, the present study performs a meta-analysis of published randomized controlled trials (RCTs) investigating acupuncture for myocardial I/R injury, providing a reference for the design and implementation of future clinical research in this field.

## 2. Materials and Methods

This study adheres to the Preferred Reporting Items for Systematic Reviews and Meta-Analyses (PRISMA) statement [22] and the Assessing the Methodological Quality of Systematic Reviews (AMSTAR) guidelines [23].

### 2.1. Inclusion Criteria and Exclusion Criteria of the Research Literature

#### 2.1.1. Inclusion Criteria of the Research Literature

1. Study Design: This includes RCTs evaluating acupuncture for myocardial I/R injury.
2. Participants: It includes patients diagnosed with myocardial I/R injury, including those undergoing coronary angioplasty, thrombolytic therapy for myocardial infarction, or cardiopulmonary bypass surgery, irrespective of age, sex, nationality, or ethnicity.
3. Interventions: The experimental group received acupuncture-based treatments, including electroacupuncture, transcutaneous electrical acupoint stimulation, auricular therapy, or acupuncture in combination with other interventions. The control group underwent sham acupuncture, standard pharmacotherapy, or nonacupuncture-related treatments. If the experimental group received acupuncture alongside other therapies, the control group was required to follow an identical treatment regimen, excluding acupuncture.
4. Treatment Timing: Acupuncture was administered either pre- or postanesthesia induction.
5. Treatment Parameters: No restrictions were imposed on treatment duration, stimulation intensity, or acupoint selection.
6. Outcome Measures: This includes the following:
  a. Serum myocardial enzyme levels: These levels include cTnI, creatine kinase-myoglobin binding force (CK-MB), creatine kinase (CK), aspartate aminotransferase (AST), and lactate dehydrogenase (LDH).
  b. Inflammatory markers: These include high sensitivity (hs)-CRP, tumor necrosis factor-α (TNF-α), interleukin-10 (IL-10), IL-6, IL-2, and IL-1.
  c. Oxidative stress indicators: These include malondialdehyde (MDA) and superoxide dismutase (SOD).
  d. Echocardiographic parameters: These include left ventricular ejection fraction (LVEF), left ventricular shortening rate (LVFS), left ventricular end-diastolic diameter (LVEDD), left ventricular end-diastolic volume (LVEDV), left ventricular end-systolic volume (LVESV), and left ventricular end-systolic diameter (LVESD).
  e. Cardiac functional recovery: This includes heartbeat recovery rate.
  f. TCM symptom score: This score includes chest pain severity assessment. Studies were included if they reported at least one of the above outcome measures.
7. Language Restrictions: Only studies published in Chinese or English were considered.

#### 2.1.2. Exclusion Criteria of the Research Literature

1. Study Type: Semi-RCTs, randomized crossover trials, case reports, research protocols, animal studies, theoretical discussion, expert reviews, or systematic reviews were excluded.
2. Interventions: Studies unrelated to acupuncture or those comparing different acupuncture modalities without a control group were not included.
3. Participants: Studies that did not involve myocardial I/R injury patients or that assessed unrelated biomarkers were excluded.
4. Duplicate Publications: Repeated studies were omitted.
5. Data Integrity: Articles with incomplete data, significant statistical errors, or methodological flaws that compromised the validity of conclusions were excluded.

### 2.2. Data Retrieval

Computerized searches were conducted across the following databases from inception to November 3, 2024: PubMed, Web of Science, Cochrane Library, Chinese National Knowledge Infrastructure (CNKI), Wanfang Database, and China Science and Technology Journal Database (VIP). Additionally, reference lists of included studies were screened to identify the supplementary relevant literature. The search strategy combined controlled vocabulary with free-text terms. Chinese search terms included (“针灸” OR “针刺” OR “艾灸” OR “电针” OR “毫针” OR “头针” OR “皮内针” OR “经皮穴位电刺激” OR “耳穴” OR “温针灸”) AND (“心肌缺血再灌注损伤” OR “心肌缺血再灌注” OR “心脏置换” OR “心脏溶栓” OR “PCI术后” OR “心肌I/R” OR “心脏手术” OR “经皮冠脉介入” OR “心脏支架” OR “MIRI”). English search terms included (“Electroacupuncture” OR “Acupuncture” OR “Moxibustion” OR “Auricular acupuncture” OR “Acupoint” OR “Transcutaneous electrical acupoint stimulation” OR “TEAS”) AND (“MI/R injury” OR “MIRI” OR “Myocardial I/R” OR “Myocardial ischemia-reperfusion injury” OR “Myocardial reperfusion injury” OR “Cardiac replacement” OR “Cardiac thrombolysis” OR “PCI” OR “Heart surgery” OR “Percutaneous coronary intervention” OR “Cardiac stent”). Furthermore, the Chinese Clinical Trial Registry and ClinicalTrials.gov (https://clinicaltrials.gov/) were queried to identify ongoing or recently completed studies. The specific search strategy for PubMed is detailed in Table 1.

### 2.3. Outcome Assessment Indicators

Primary outcome measures included the following: (1) serum myocardial enzyme levels (cTnI, CK-MB, CK, AST, and LDH); (2) serum inflammatory markers (hs-CRP, TNF-α, IL-10, IL-6, IL-2, and IL-1); (3) serum oxidative stress markers (MDA and SOD); (4) echocardiographic parameters (LVEF, LVFS, LVEDD, LVEDV, LVESV, and LVESD); (5) heartbeat recovery rate; and (6) TCM chest pain symptom score. Secondary outcomes comprised the following: (1) ICU length of stay, (2) total hospital length of stay, and (3) incidence of major adverse cardiovascular events (MACE).

### 2.4. Literature Screening and Data Extraction

Two researchers independently screened the literature, extracted data, and conducted cross-verification based on the specified criteria. Literature selection was managed using EndNote X9. The initial screening involved reviewing titles and abstracts to exclude irrelevant studies, followed by a full-text assessment to determine eligibility. Extracted data were systematically tabulated, including study title, author, sample size, patient age, intervention details, intervention timing, and all reported outcomes. Any discrepancies were resolved through discussion, with arbitration by a third researcher if needed.

### 2.5. Risk Bias and Quality Assessment

Methodological quality was assessed using Review Manager 5.3, following the Cochrane Collaboration's risk-of-bias framework, which evaluates seven domains: random sequence generation (selection bias), allocation concealment (selection bias), blinding of participants and personnel (performance bias), blinding of outcome assessors (detection bias), completeness of outcome data (attrition bias), selective outcome reporting (reporting bias), and other potential sources of bias. Two reviewers independently rated each study as having a low, unclear, or high risk of bias and cross-checked the results. Discrepancies were resolved through discussion, with a third reviewer consulted if necessary. Additionally, the GRADEpro Guideline Development Tool (GDT) was used to evaluate the quality of evidence.

### 2.6. Statistical Treatment

To obtain original data for graphical results, the corresponding author was initially contacted. If data remained unavailable, Web Plot Digitizer was used to extract Mean ± SD values from images. RevMan 5.3 and Stata 16 were employed for statistical analysis, including meta-analysis and effect size estimation. Meta-analysis results were presented via pairwise comparison of tables and forest plots. For single-target indicators, results were also reported based on normative effects. For continuous variables, the mean difference (MD) was used to calculate effect sizes, while the standardized mean difference (SMD) was applied to mitigate heterogeneity due to variations in measurement units and methods. For categorical data, the relative risk (RR) with a 95% confidence interval (CI) was computed. A heterogeneity threshold of *p*=0.1 was set, with *p* < 0.1 indicating significant heterogeneity. The *I*^2^ statistic was used for quantitative heterogeneity assessment, with *I*^2^ > 50% indicating substantial heterogeneity. In such cases, a random effects model (REM) was applied; otherwise, a fixed effects model (FEM) was used. When significant heterogeneity was detected, a subgroup analysis or sensitivity analysis was conducted based on the timing of outcome measurement to identify potential sources of variation. Publication bias was assessed for outcome indicators with more than 10 studies using funnel plots. The Egger test was performed to detect publication bias, with *p* < 0.1 considered statistically significant. If substantial heterogeneity persisted, descriptive analysis was conducted, and sensitivity analysis was performed to assess the robustness of the findings.

## 3. Results

### 3.1. Literature Screening Process and Results

**Figure 1:**
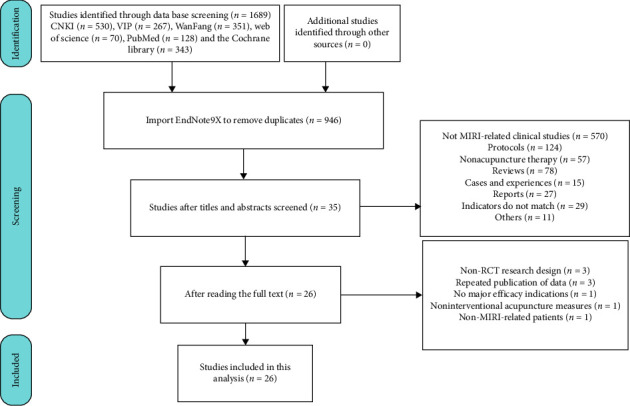
Flowchart illustrating the literature screening process.

A total of 1689 relevant studies were retrieved, including 530 from CNKI, 267 from VIP, 351 from Wanfang, 70 from Web of Science, 128 from PubMed, and 343 from the Cochrane Library. Studies were screened based on predefined inclusion and exclusion criteria, yielding 946 unique records after removing duplicates. Following title and abstract screening, exclusions included 391 animal studies, 179 unrelated to nonmyocardial I/R injury, 121 Cochrane Library registers, three study protocols, 57 nonacupuncture studies, 78 reviews, 15 case reports, 11 news reports, four announcements, one advertisement, 11 clinical study reports, and 29 clinical observations. Additional exclusions comprised five theoretical discussion, two letters, two editorials, one questionnaire survey, and one clinical application guideline. Full-text analysis of 35 shortlisted articles led to the exclusion of three non-RCT designs, three duplicate data publications, one study lacking key efficacy indicators for myocardial I/R injury, one unrelated to acupuncture treatment, and one involving nonpatient subjects. Ultimately, 26 studies met the eligibility criteria and were included in the meta-analysis [20, 21, 24–47]. Representative exclusions included a study [16] demonstrating that acupuncture significantly reduced angina frequency and severity while improving 6-min walk test (6MWT) performance. However, its inclusion of coronary heart disease or stent-implantation patients, along with discrepancies in reported indicators, rendered it ineligible. The study selection process is illustrated in Figure 1.

### 3.2. Basic Characteristics and Overview of Included Studies

Twenty-six RCTs conducted between 1999 and the present have enrolled a total of 1948 patients, aged 2–80 years, undergoing cardiac surgery. Among them, 970 patients in the intervention group received acupuncture, electroacupuncture, or transcutaneous electrical stimulation at acupuncture points, while 978 in the control group underwent sham acupuncture, sham electroacupuncture, sham transcutaneous electrical stimulation, or no intervention. Baseline characteristics were comparable across all studies. Notably, all trials were conducted in China, with four published in English-language databases and 22 in Chinese databases.  Table 2 provides a detailed summary of study characteristics.

### 3.3. Method Quality Assessment of Included Studies

**Figure 2:**
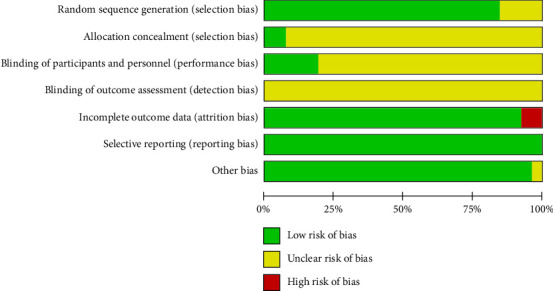
Overall risk bias assessment for included studies.

**Figure 3:**
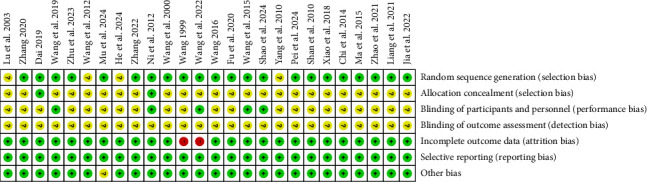
Individual risk bias assessment chart for included studies.

The risk assessment tool from the Cochrane Handbook was used to evaluate the included studies, indicating a generally moderate risk across most literature studies. All 26 studies were RCTs, with 18 [25–36, 39–41, 43, 45, 47] employing the random number table method, two [24, 37] utilizing computer-generated randomization, two [21, 38] implementing envelope randomization, and four [20, 42, 44, 46] mentioning randomization without specifying the method. Regarding allocation concealment, only two studies [21, 24] explicitly described their concealment strategy. In terms of blinding, five studies [21, 29, 30, 37, 47] reported blinding both researchers and participants, while none of the 16 studies mentioned blinding of outcome assessors. Data completeness issues were noted in two studies [31, 32]. For reporting bias, all 26 studies adhered to their prespecified outcome reporting. No additional biases were detected, as baseline characteristics were comparable across all studies. A detailed risk of bias assessment is presented in Figures 2 and 3.

### 3.4. Meta-Analysis Results of Acupuncture Treatment of Myocardial I/R Injury

#### 3.4.1. Myocardial Enzyme Level

##### 3.4.1.1. cTnI

Eight studies [20, 21, 24, 26, 28, 30, 37, 39] reported cardiac troponin I (cTnI) levels. One study [37] converted measurement data into categorical variables, precluding statistical extraction. Substantial heterogeneity arose due to variations in study design and detection timing, necessitating combined and subgroup analyses. Meta-analysis revealed significant differences in cTnI levels at 0.5, 2, 3, 6, 8, and 24 h between the acupuncture and control groups, with acupuncture significantly reducing cTnI levels. However, no significant differences were observed at 0.25, 1, 12, 48, or 72 h. The overall combined effect size for cTnI was (SMD = −1.22, 95% CI [−1.57, −0.87]), *Z* = 6.77, and *p* < 0.00001, confirming a statistically significant reduction in myocardial enzyme levels with acupuncture. Notably, acupuncture exhibited a “parabolic” trend, predominantly mitigating peak cTnI elevation postmyocardial injury, as detailed in Table 3 and Supporting Figure 1.

##### 3.4.1.2. CK-MB

Five studies [24, 26, 27, 41, 43] reported CK-MB levels. Given variations in study design and detection timing, a meta-analysis and a subgroup analysis were performed. Heterogeneity among the studies was minimal. The meta-analysis revealed a significant reduction in CK-MB levels at 6, 12, 24, and 48 h and 14 days in the acupuncture group compared to controls. However, no significant differences were observed at 72 h or 7 days. The overall combined effect size for CK-MB was (SMD = −0.71, 95% CI [−0.87, −0.55]), *Z* = 8.63, and *p* < 0.00001, confirming a statistically significant reduction in CK-MB levels with acupuncture. These findings indicate that acupuncture effectively lowers myocardial CK-MB levels in patients with myocardial I/R injury, as detailed in Table 4 and Supporting Figure 2.

##### 3.4.1.3. CK

**Figure 4:**
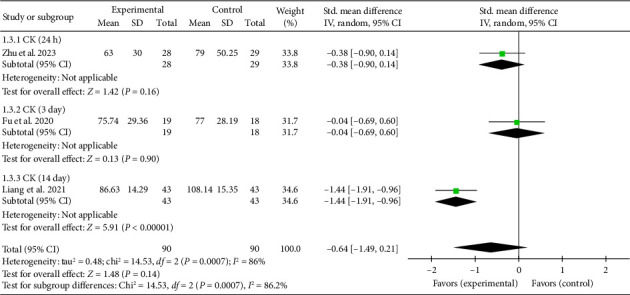
Forest plot of myocardial enzyme CK in acupuncture and control groups.

Three studies [26, 27, 43] reported myocardial enzyme CK levels. The overall combined effect size was (SMD = −0.64, 95% CI [−1.49, 0.21]), *Z* = 1.48, and *p*=0.14, indicating no statistically significant effect of acupuncture on CK levels in patients with myocardial I/R injury. The forest plot illustrating CK levels is presented in Figure 4.

##### 3.4.1.4. AST

**Figure 5:**
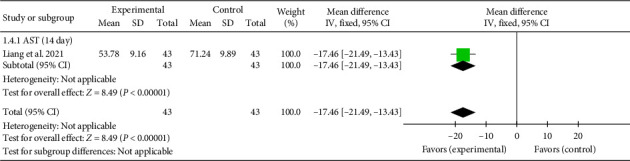
Forest plot of myocardial enzyme AST in acupuncture and control groups.

A single study [27] reported AST levels on Day 14. The aggregated effect size was (MD = −17.46, 95% CI [−21.49, −13.43]), *Z* = 8.49, and *p* < 0.00001, demonstrating a significant reduction in AST levels with acupuncture in patients with myocardial I/R injury. The forest plot for AST levels is shown in Figure 5.

##### 3.4.1.5. LDH

**Figure 6:**
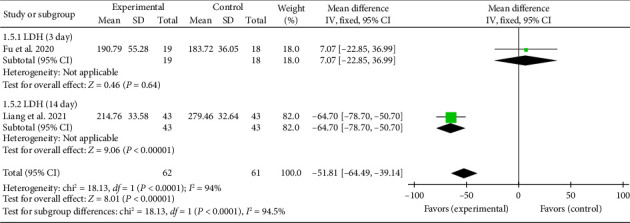
Forest plot of myocardial enzyme CK in acupuncture and control groups.

Two studies [27, 43] reported LDH levels on Days 3 and 14. One study [27] found a significant reduction in LDH levels at Day 14 (MD = −64.70, 95% CI [−78.70, −50.70]), *Z* = 9.06, and *p* < 0.00001, indicating a significant difference between the acupuncture and control groups. In contrast, another study [43] reported no significant difference in LDH levels at Day 3 (MD = 7.07, 95% CI [−22.85, 36.99]), *Z* = 0.46, and *p*=0.64. The forest plot for LDH levels is presented in Figure 6.

##### 3.4.1.6. BNP

Two studies [31, 41] reported BNP levels. Due to variations in study design and detection timing, substantial heterogeneity was observed, necessitating combined and subgroup analyses. The meta-analysis revealed significant reductions in BNP levels at Days 1, 3, 7, and 14 in the acupuncture group compared to controls. The overall combined effect size for BNP was (SMD = −2.89, 95% CI [−4.95, −0.83]), *Z* = 2.75, and *p*=0.006, confirming a statistically significant reduction in BNP levels with acupuncture. These findings indicate that acupuncture effectively lowers BNP levels in patients with myocardial I/R injury, as detailed in Table 5 and Supporting Figure 3.

#### 3.4.2. Inflammatory Factor Level

##### 3.4.2.1. hs-CRP

Seven studies [21, 24, 34, 35, 37–39] reported hs-CRP levels. Due to variations in study design and detection timing, substantial heterogeneity was observed, necessitating a meta-analysis and subgroup analysis. The meta-analysis revealed significant reductions in hs-CRP levels at 0.5, 2, 8, 12, and 24 h and 30 days in the acupuncture group compared to controls. However, no significant differences were observed at 6, 48, and 72 h. The overall combined effect size for hs-CRP was (SMD = −1.34, 95% CI [−1.87, −0.80]), *Z* = 4.92, and *p* < 0.00001, confirming a statistically significant reduction in hs-CRP levels with acupuncture. These findings indicate that acupuncture effectively reduces hs-CRP levels in patients with myocardial I/R injury, as detailed in Table 6 and Supporting Figure 4.

##### 3.4.2.2. TNF-α

Four studies [21, 25, 29, 37] reported TNF-α levels, exhibiting substantial heterogeneity due to variations in study design and detection timing. To address this, a meta-analysis and a subgroup analysis were conducted. The meta-analysis revealed a significant reduction in TNF-α levels at 0, 0.5, 6, 24, and 72 h and 14 days in the acupuncture group compared to controls. However, no significant difference was observed at 2 h. The overall combined effect size for TNF-α was (SMD = −1.13, 95% CI [−1.41, −0.85]), *Z* = 7.84, and *p* < 0.00001, confirming a statistically significant reduction in TNF-α levels with acupuncture. These findings suggest that acupuncture effectively lowers TNF-α levels in patients with myocardial I/R injury, as presented in Table 7 and Supporting Figure 5.

##### 3.4.2.3. IL-10

Five studies [21, 25, 29, 35, 37] reported IL-10 levels, exhibiting substantial heterogeneity due to variations in study design and detection timepoints. To account for this, a meta-analysis and a subgroup analysis were conducted. The analysis revealed a significant increase in IL-10 levels at 0, 0.5, 6, 8, 24, and 72 h in the acupuncture group compared to controls. However, no significant difference was observed at 2 h. The overall combined effect size for IL-10 was (SMD = 1.31, 95% CI [0.74, 1.87]), *Z* = 4.55, and *p* < 0.00001, confirming a statistically significant elevation in IL-10 levels with acupuncture. These findings suggest that acupuncture effectively enhances IL-10 levels in patients with myocardial I/R injury, as detailed in Table 8 and Supporting Figure 6.

##### 3.4.2.4. IL-6

Seven studies [21, 29, 34, 35, 37, 38, 45] reported IL-6 levels, with substantial heterogeneity due to variations in study design and detection timepoints. To address this, a meta-analysis and a subgroup analysis were conducted. The meta-analysis revealed significant reductions in IL-6 levels at 0, 0.5, 2, 8, and 24 h and 30 days in the acupuncture group compared to controls. Acupuncture did not demonstrate a significant reduction in IL-6 levels at the 6 and 72-h time points, as no statistically significant differences were observed between the acupuncture and control groups. The overall combined effect size for IL-6 was (SMD = −1.53, 95% CI [−2.01, −1.06]), *Z* = 6.33, and *p* < 0.00001, confirming a statistically significant reduction in IL-6 levels with acupuncture. These findings highlight the efficacy of acupuncture in lowering IL-6 levels in patients with myocardial I/R injury, as detailed in Table 9 and Supporting Figure 7.

##### 3.4.2.5. IL-2

**Figure 7:**
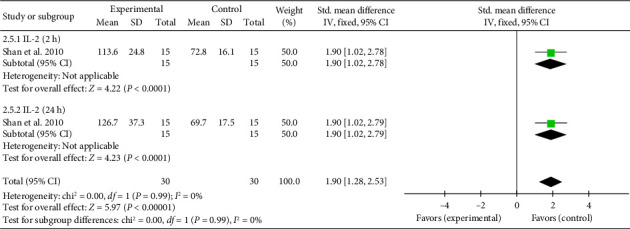
Forest plot of the inflammatory factor IL-2 in acupuncture and control groups.

A single study [25] reported IL-2 levels, exhibiting significant heterogeneity due to differences in study design and detection timepoints. To account for this, a meta-analysis and a subgroup analysis were conducted. The combined effect size for IL-2 levels at 2 h was (SMD = 1.90, 95% CI [1.02, 2.78]), *Z* = 4.22, and *p* < 0.0001, indicating a significant increase in IL-2 levels with acupuncture. Similarly, at 24 h, the combined effect size was (SMD = 1.90, 95% CI [1.02, 2.79]), *Z* = 4.23, and *p* < 0.0001, confirming a statistically significant elevation in IL-2 levels in patients with myocardial I/R injury. The forest plot for IL-2 levels is presented in Figure 7.

##### 3.4.2.6. IL-1

**Figure 8:**
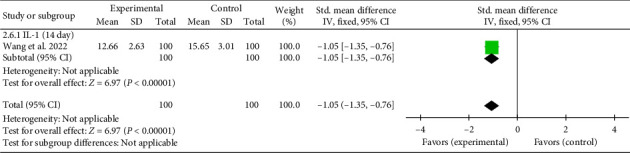
Forest plot of the inflammatory factor IL-1 in acupuncture and control groups.

A single study [31] reported IL-1 levels on Day 14. The combined effect size was (SMD = −1.05, 95% CI [−1.35, −0.76]), *Z* = 6.97, and *p* < 0.00001, indicating a statistically significant reduction in IL-1 levels with acupuncture in patients with myocardial I/R injury. The forest plot for IL-1 levels is presented in Figure 8.

##### 3.4.2.7. IL-8

**Figure 9:**
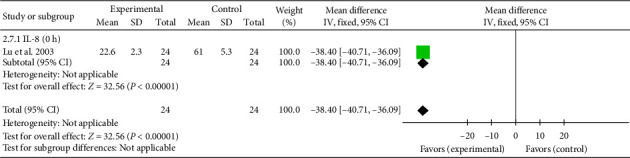
Forest plot of the inflammatory factor IL-1 in acupuncture and control groups.

A single study [42] reported IL-8 levels at 0 h. The effect size was (MD = −38.40, 95% CI [−40.71, −36.09]), *Z* = 32.56, and *p* < 0.00001, indicating a statistically significant reduction in IL-8 levels with acupuncture in patients with myocardial I/R injury. The forest plot for IL-8 levels is presented in Figure 9

#### 3.4.3. Oxidative Stress Factor Levels

##### 3.4.3.1. MDA

Five studies [28, 30, 33, 42, 46] reported MDA levels, exhibiting considerable heterogeneity due to variations in study design and detection timepoints. To address this, a meta-analysis and a subgroup analysis were conducted. The meta-analysis revealed significant reductions in MDA levels at 0, 0.5, 1, 2, 6, and 24 h in the acupuncture group compared to controls, while no significant difference was observed at 0.25 h. The overall combined effect size for MDA was (SMD = −1.78, 95% CI [−2.36, −1.20]), *Z* = 6.00, and *p* < 0.00001, confirming a statistically significant reduction in MDA levels with acupuncture. These findings highlight the efficacy of acupuncture in mitigating oxidative stress in patients with myocardial I/R injury, as detailed in Table 10 and Supporting Figure 8.

##### 3.4.3.2. SOD

Four studies [30, 33, 42, 46] reported SOD levels, exhibiting substantial heterogeneity due to differences in study design and detection timepoints. To address this, a meta-analysis and a subgroup analysis were conducted. The meta-analysis revealed significant differences in SOD levels at 0, 0.5, 1, 6, and 24 h, with acupuncture significantly increasing SOD levels compared to the control group. However, no significant difference was observed at 0.25 h. The overall combined effect size for SOD was (SMD = 0.98, 95% CI [0.55, 1.41]), *Z* = 4.50, and *p* < 0.00001, confirming a statistically significant enhancement of SOD levels with acupuncture. These findings suggest that acupuncture effectively promotes antioxidant activity in patients with myocardial I/R injury, as detailed in Table 11 and Supporting Figure 9.

#### 3.4.4. Echocardiogram Indexes

##### 3.4.4.1. LVEF

Eight studies [27, 31, 37, 39–41, 44, 47] reported echocardiographic LVEF. Given the heterogeneity arising from variations in study design and assessment time points, both meta-analysis and subgroup analysis were conducted. The meta-analysis and effect size evaluation revealed a significant difference in echocardiographic LVEF between the acupuncture and control groups at Days 0, 7, 14, 28, 42, 56, 90, and 180. Acupuncture was associated with a significant improvement in LVEF compared to the control group at these time points, except at Day 5, where no statistically significant difference was observed. The overall pooled effect size for echocardiographic LVEF was SMD = 1.32 (95% CI [0.54, 2.09]), *Z* = 3.33, and *p*=0.0009, indicating a significant enhancement in LVEF in patients with myocardial I/R injury in the acupuncture group, as detailed in Table 12 and Supporting Figure 10.

##### 3.4.4.2. LVFS

**Figure 10:**
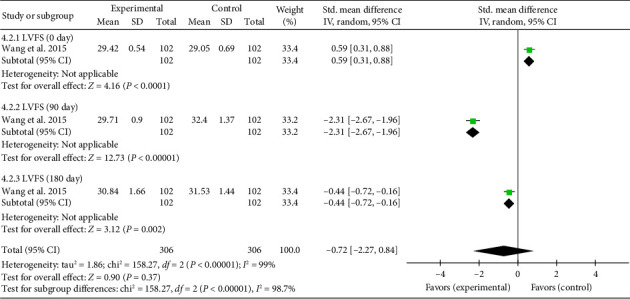
Forest plot of the LVFS in acupuncture and control groups.

A single study [37] reported LVFS assessed by echocardiography. Due to heterogeneity arising from variations in study design and detection timepoints, a meta-analysis and a subgroup analysis were conducted. The analysis revealed a significant increase in LVFS at Day 0 (SMD = 0.59, 95% CI [0.31, 0.88]), *Z* = 4.16, and *p* < 0.0001, indicating an initial improvement in LVFS with acupuncture in patients with myocardial I/R injury. However, at Day 90, LVFS significantly decreased in the acupuncture group compared to controls (SMD = −2.31, 95% CI [−2.67, −1.96]), *Z* = 12.73, and *p* < 0.00001. A similar trend was observed at Day 180 (SMD = −0.44, 95% CI [−0.72, −0.16]), *Z* = 3.12, and *p*=0.002, demonstrating a sustained reduction in LVFS over time. These findings suggest a time-dependent effect of acupuncture on LVFS in myocardial I/R injury. The forest plot for LVFS is presented in Figure 10.

##### 3.4.4.3. LVEDD

Echocardiographic LVEDD measurements were reported in three studies [27, 37, 44]. Due to variations in study design and assessment timepoints, significant heterogeneity was observed, necessitating meta-analysis and subgroup analysis. One study reported a combined effect size for LVEDD at day 0 (SMD = 0.50, 95% CI [0.22, 0.78], *Z* = 3.49, *p*=0.0005), indicating that acupuncture significantly increased LVEDD in myocardial I/R injury. Another study reported a combined effect size at Day 14 (SMD = −0.72, 95% CI [−1.16, −0.28], *Z* = 3.23, and *p*=0.001), suggesting that acupuncture significantly reduced LVEDD. A third study found a combined effect size at Day 28 (SMD = −1.01, 95% CI [−1.49, −0.53], *Z* = 4.13, and *p* < 0.0001), indicating that acupuncture significantly increased LVEDD conditions. Additionally, a study reported a combined effect size at Day 90 (SMD = 2.04, 95% CI [1.70, 2.37], *Z* = 11.76, and *p* < 0.00001), suggesting a significant increase in LVEDD. Finally, a study reported a combined effect size at Day 180 (SMD = −3.64, 95% CI [−4.09, −3.19], *Z* = 15.85, and *p* < 0.00001), suggesting a significant reduction in LVEDD in myocardial I/R injury, as detailed in Table 13 and Supporting Figure 11.

##### 3.4.4.4. LVEDV

Two studies [37, 41] assessed echocardiographic LVEDV. At Day 0, the pooled effect size (SMD = 0.85, 95% CI [0.57, 1.14], *Z* = 5.82, and *p* < 0.00001) indicated a significant increase in LVEDV following acupuncture in myocardial I/R injury cases. However, at Day 7 (SMD = 0.06, 95% CI [−0.36, 0.48], *Z* = 0.29, and *p*=0.77) and Day 90 (SMD = −0.05, 95% CI [−0.33, 0.22], *Z* = 0.38, and *p*=0.71), no significant differences were observed between the acupuncture and control groups. By Day 180, the pooled effect size (SMD = −2.86, 95% CI [−3.25, −2.46], *Z* = 14.28, and *p* < 0.00001) demonstrated a significant reduction in LVEDV with acupuncture, as presented in Table 14 and Supporting Figure 12.

##### 3.4.4.5. LVESV

Two studies [37, 41] evaluated echocardiographic LVESV. At Day 0, the pooled effect size (SMD = 1.10, 95% CI [0.80, 1.39], *Z* = 7.30, and *p* < 0.00001) indicated a significant increase in LVESV following acupuncture in myocardial I/R injury cases. However, at Day 7 (SMD = 0.31, 95% CI [−0.11, 0.73], *Z* = 1.43, and *p*=0.15) and Day 90 (SMD = 0.22, 95% CI [−0.05, 0.50], *Z* = 1.57, and *p*=0.12), no significant differences were observed between the acupuncture and control groups. By Day 180, the pooled effect size (SMD = 3.56, 95% CI [3.12, 4.01], *Z* = 15.72, and *p* < 0.00001) demonstrated a substantial increase in LVESV with acupuncture, as presented in Table 15 and Supporting Figure 13.

##### 3.4.4.6. LVESD

**Figure 11:**
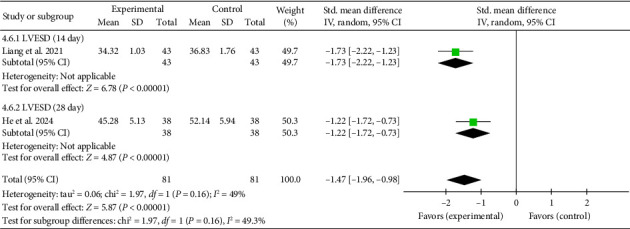
Forest plot of the LVESD in acupuncture and control groups.

One study [27] assessed echocardiographic LVESD. At Day 14, the pooled effect size (SMD = −1.73, 95% CI [−2.22, −1.23], *Z* = 6.78, and *p* < 0.00001) indicated a significant reduction in LVESD with acupuncture. Similarly [44], at Day 28, the pooled effect size (SMD = −1.22, 95% CI [−1.72, −0.73], *Z* = 4.87, and *p* < 0.00001) confirmed a continued decrease. These findings suggest that acupuncture effectively reduced LVESD at both time points in myocardial I/R injury cases. The forest plot for LVESD is presented in Figure 11.

#### 3.4.5. Heartbeat Recovery Rate

**Figure 12:**
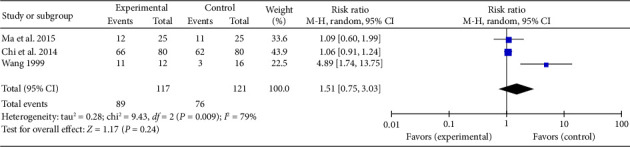
Forest plot of heartbeat recovery rate in acupuncture and control groups.

Three studies [30, 32, 36] reported a combined effect size for the heartbeat recovery rate in myocardial I/R injury cases (RR = 1.51, 95% CI [0.75, 3.03], *Z* = 1.17, and *p*=0.24), indicating no significant improvement with acupuncture. The forest plot for heartbeat recovery rate is presented in Figure 12.

#### 3.4.6. TCM Chest Pain Symptom Score

**Figure 13:**
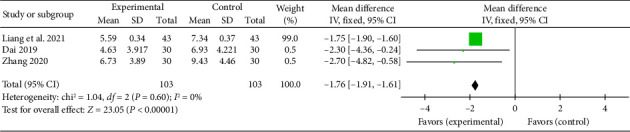
Forest plot of TCM chest pain symptom scores in the acupuncture and control groups.

Three studies [24, 27, 34] reported a pooled effect size for the TCM chest pain symptom score (MD = −1.76, 95% CI [−1.91, −1.61], *Z* = 23.05, and *p* < 0.00001), indicating a significant reduction in chest pain symptoms with acupuncture in myocardial I/R injury cases. The forest plot for TCM chest pain symptom scores is presented in Figure 13.

#### 3.4.7. Duration of ICU Stay

**Figure 14:**
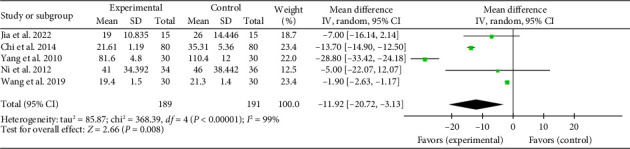
Forest plot of the duration of ICU stay in the acupuncture and control groups.

Five studies [20, 21, 29, 36, 45] reported a pooled effect size for ICU stay duration (MD = −11.92, 95% CI [−20.72, −3.13], *Z* = 2.66, and *p*=0.008), indicating a significant reduction with acupuncture in myocardial I/R injury cases. The forest plot for ICU stay duration is presented in Figure 14.

#### 3.4.8. Duration of Hospital Stay

**Figure 15:**
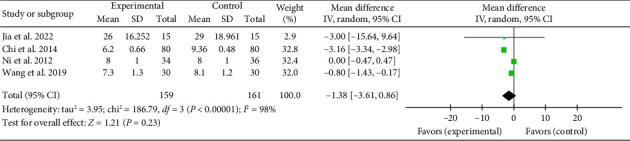
Forest plot of duration of hospital stay in the acupuncture and control groups.

Four studies [21, 29, 36, 45] reported a combined effect size for hospital stay duration (MD = −1.38, 95% CI [−3.61, 0.86], *Z* = 1.21, and *p*=0.23), indicating no significant reduction with acupuncture in myocardial I/R injury cases. The forest plot for hospital stay duration is presented in Figure 15.

#### 3.4.9. Incidence of MACE

**Figure 16:**
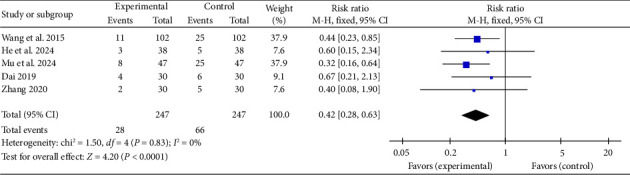
Forest plot of MACE incidence in acupuncture and control groups.

Five studies [24, 34, 37, 39, 44] reported a pooled effect size for MACE incidence (RR = 0.42, 95% CI [0.28, 0.63], *Z* = 4.20, and *p* < 0.0001), demonstrating a significant reduction in MACE occurrence with acupuncture in myocardial I/R injury cases. The forest plot for MACE incidence is presented in Figure 16.

#### 3.4.10. 6MWT

**Figure 17:**
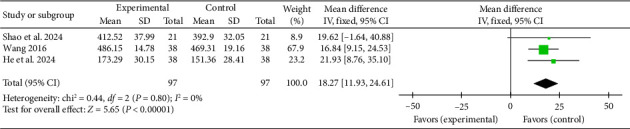
Forest plot of MACE incidence in acupuncture and control groups.

Three studies [40, 44, 47] reported a pooled effect size for the 6MWT (MD = 18.27, 95% CI [11.93, 24.61], *Z* = 5.65, and *p* < 0.00001), indicating a significant improvement in exercise capacity with acupuncture in myocardial I/R injury cases. The forest plot for the 6MWT is presented in Figure 17.

#### 3.4.11. SAQ

**Figure 18:**
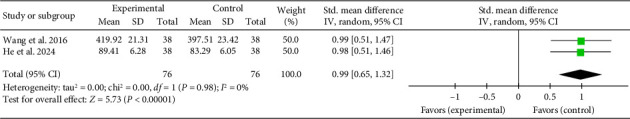
Forest plot of SAQ incidence in acupuncture and control groups.

Two studies [40, 44] reported a combined effect size for the SAQ (SMD = 0.99, 95% CI [0.65, 1.32], *Z* = 5.73, and *p* < 0.00001), indicating a significant improvement in SAQ scores with acupuncture in myocardial I/R injury cases. The forest plot for SAQ is presented in Figure 18.

### 3.5. Assessment of Publication Bias

No publication bias analysis was performed, as none of the included outcome indicators were supported by more than 10 studies.

### 3.6. Security

None of the 26 studies reported adverse effects associated with acupuncture therapy, suggesting a favorable safety profile for acupuncture in myocardial I/R injury.

### 3.7. Evaluation of Evidence Quality

Evidence quality, assessed using the GRADE system, ranged from moderate to very low. The incidence of MACE was rated as moderate quality, while AST, IL-2, IL-1, IL-8, LVESD, TCM chest pain symptom score, 6MWT, and SAQ were classified as low quality. The evidence for cTnI, CK-MB, CK, LDH, BNP, hs-CRP, TNF-α, IL-10, IL-6, MDA, SOD, LVEF, LVFS, LVEDD, LVEDV, LVESV, heartbeat recovery rate, ICU stay duration, and hospital stay duration was rated as very low quality, as detailed in Table 16.

## 4. Discussion

Myocardial I/R injury is a contemporary pathological concept characterized by exacerbated myocardial tissue damage upon the restoration of blood flow to previously occluded coronary arteries. This process is intricately linked to postoperative complications associated with coronary angioplasty, coronary revascularization, and heart transplantation. In 1960, Jennings et al. [48] first reported in a canine myocardial ischemia model that ischemic myocardial tissue exhibits exacerbated necrotic damage following reperfusion. Subsequently, in 1985, Braunwald and Kloner [49] further refined the concept of “myocardial ischemia-reperfusion injury.” Clinically, myocardial I/R injury manifests in several forms: (1) myocardial stunning, also referred to as post-ischemic cardiac insufficiency, characterized by transient systolic and diastolic dysfunction postreperfusion; (2) reperfusion arrhythmias, including ventricular tachycardia and ventricular fibrillation; (3) microcirculatory disturbances, attributed to microvascular obstruction and myocardial hemorrhage; (4) the no-reflow phenomenon, marked by impaired or absent restoration of coronary blood flow postreperfusion therapy, leading to inadequate tissue perfusion; and (5) lethal myocardial reperfusion injury. Given the high incidence of myocardial reperfusion injury following cardiac surgery, effective strategies for its prevention and management remain a critical focus in acute myocardial infarction and cardiac surgery treatment. While the precise pathogenesis of myocardial I/R injury remains elusive, three key pathological mechanisms have been identified: energy metabolism dysregulation and calcium overload serve as primary triggers [50, 51], oxidative stress and inflammatory responses are indispensable contributors [52, 53], and various forms of cell death ultimately determine the cardiac outcome [54].

Acupuncture, a cornerstone of traditional Oriental medicine with a history spanning millennia, has gained recognition from the WHO for its therapeutic efficacy in 107 conditions [55], including cardiovascular disorders such as coronary artery disease, angina, hypertension, arrhythmia, chronic pulmonary heart disease, and cardiac insufficiency. Acupuncture offers several advantages in managing myocardial I/R injury. As a nonpharmacological intervention, it provides a safer and more cost-effective alternative without the risks of drug dependency or adverse effects. Moreover, acupuncture serves as a valuable tool for investigating cardiac function and the mechanisms underlying various therapeutic interventions. Experimental studies have demonstrated its capacity to modulate myocardial electrical activity, cardiovascular microcirculation, cytokine levels, epigenetic modifications, and central regulatory mechanisms, thereby enhancing cardiac function. Notably, acupuncture exerts cardioprotective effects in myocardial disorders associated with calcium overload, energy metabolism dysregulation, mitophagy, apoptosis, oxidative stress, inflammatory responses, and related signaling pathways [56–61]. Systematic reviews of evidence-based experimental research further support electroacupuncture's protective role in myocardial I/R injury animal models [62].

This study analyzed 26 clinical trials investigating acupuncture for myocardial I/R injury, encompassing 1948 patients. All trials, comprising 22 Chinese and four English publications, were conducted in China. The treatment group primarily received acupuncture and moxibustion, with 15 studies employing electroacupuncture, six using traditional acupuncture, and four utilizing transcutaneous electrical acupoint stimulation. In the control group, 18 studies applied nonacupuncture interventions, while four used sham electroacupuncture, one employed sham acupuncture, and three implemented sham transcutaneous electrical acupoint stimulation.

Outcome measures included myocardial enzyme levels (cTnI, CK-MB, CK, AST, LDH, and BNP), serum inflammatory markers (hs-CRP, TNF-α, IL-10, IL-6, IL-2, IL-1, and IL-8), oxidative stress markers (MDA and SOD), echocardiographic parameters (LVEF, LVFS, LVEDD, LVEDV, LVESV, and LVESD), heart rate recovery, TCM-based chest pain scores, ICU and hospitalization duration, MACE incidence, 6MWT, and SAQ. Meta-analysis of the published evidence indicated that acupuncture significantly reduced myocardial enzyme levels (cTnI, CK-MB, CK, AST, and LDH) in patients with myocardial I/R injury. Inflammatory markers, including hs-CRP, TNF-α, IL-6, IL-8, and IL-1, were attenuated, whereas IL-10 and IL-2 levels increased. Additionally, oxidative stress markers MDA and SOD were reduced. Echocardiographic assessment demonstrated an increase in LVEF and LVESV, alongside reductions in LVFS, LVEDD, LVEDV, and LVESD. Acupuncture further alleviated TCM-based chest pain scores, shortened ICU stay, lowered MACE incidence, and improved 6MWT and SAQ indicators. These findings indicate that acupuncture holds therapeutic potential for mitigating myocardial I/R injury in cardiac surgery by attenuating myocardial damage, suppressing inflammatory responses, activating anti-inflammatory and immune factors, reducing oxidative stress, enhancing cardiac function, alleviating ventricular pathological remodeling, relieving chest pain, shortening ICU stays, and minimizing cardiovascular adverse events. Assessment of methodological quality based on the Cochrane Handbook criteria determined that the overall quality of the included studies was moderate. The GRADE evidence rating system was applied to evaluate all outcome measures, revealing substantial variability in quality. Among the assessed indicators, one (2.5%) was of moderate quality, six (20.5%) were classified as low quality, and 19 (48.7%) were categorized as very low quality. The primary reasons for downgrading are methodological limitations, imprecision, and potential publication bias. Additionally, inconsistencies in myocardial I/R injury treatment criteria and variations in the timing of efficacy assessments further compromised the objective evaluation of acupuncture's therapeutic effects. Despite its potential, the current evidence is constrained by limited sample sizes and methodological quality, leading to significant uncertainty in the conclusions.

Limitations of this study: (1) The inclusion of the literature was restricted to Chinese and English sources, potentially overlooking relevant studies published in Japanese, Korean, and other languages. Additionally, certain studies lacked explicit randomization methods, introducing a risk of selection bias. (2) Given that acupuncture is an interventional procedure, achieving full blinding remains inherently challenging. This methodological limitation introduces unavoidable biases, particularly in implementation and measurement, contributing to clinical heterogeneity. (3) Variability exists among studies in acupoint selection, intervention modalities (e.g., electroacupuncture, manual acupuncture, and transcutaneous electrical acupoint stimulation), and intervention timing (e.g., post-treatment application vs. full-course intervention). However, a more detailed subgroup analysis was not performed, which may compromise result accuracy. (4) The included studies encompassed a heterogeneous patient population undergoing various types of cardiac surgery, leading to potential uncertainty regarding the therapeutic efficacy of acupuncture. (5) Bias risk assessment and evidence quality evaluation indicated that most studies exhibited a moderate risk of bias, and the overall evidence quality did not meet the threshold for high-certainty evidence. Further validation through multicenter, high-quality RCTs with larger sample sizes is warranted. (6) In this study, data extraction software Web Plot Digitizer was utilized to obtain partial data from graphical representations in four studies and derive mean ± SD values [21, 35–37].

Based on these limitations, the following recommendations are proposed for future research: (1) Clinical trials investigating acupuncture for myocardial I/R injury should adhere to rigorous methodological standards and comply with reporting guidelines such as the Consolidated Standards of Reporting Trials (CONSORT) and the Standards for Reporting Interventions in Clinical Trials of Acupuncture (STRICTA) to ensure methodological robustness. (2) Researchers should employ the GRADE system to evaluate outcome measure quality, thereby enhancing the reliability of clinical decision-making. (3) To mitigate bias, strict adherence to methodological quality control throughout the research process is essential, improving the reliability of the literature and evidence base. (4) Only one RCT in the current review reported clinical trial registration, underscoring the need for mandatory trial registration and prespecified reporting to minimize publication bias. (5) Establishing standardized clinical protocols for acupuncture treatment in myocardial I/R injury research is necessary, including explicit guidelines on procedural techniques, acupoint selection, treatment timing, and transparent criteria for concurrent medication use.

## Figures and Tables

**Table 1:** PubMed search strategy.

Number of the formula	Combination of search terms
#1	Electroacupuncture': ab,ti OR ‘acupuncture': ab,ti OR ‘moxibustion': ab,ti OR ‘auricular acupuncture': ab,ti OR ‘acupoint': ab,ti OR ‘transcutaneous electrical acupoint stimulation': ab,ti OR ‘teas': ab,ti
#2	Mi/r injury': ab,ti OR ‘miri': ab,ti OR ‘myocardial i/r': ab,ti OR ‘myocardial ischemia reperfusion injury': ab,ti OR ‘myocardial reperfusion injury': ab,ti OR ‘cardiac replacement': ab,ti OR ‘cardiac thrombolysis': ab,ti OR ‘pci': ab,ti OR ‘heart surgery': ab,ti OR ‘percutaneous coronary intervention': ab,ti OR ‘cardiac stent': ab,ti
#3	#1 AND #2

**Table 2:** Basic characteristics of included studies.

Serial number	Author	Year	Number of cases		Sex (male/female)	Age (years)	Surgical procedure	Intervention		Selection of acupuncture points	Outcome indicator	Follow-up duration	Region
			Test group	Control group				Acupuncture group	Control group				
1	Yang et al. [20]	2010	30	30	27/33	47–51	Heart valve replacement surgery	Electroacupuncture preconditioning, 5 times, once each day, 30 min each time	Sham electroacupuncture	Neiguan (PC6); Lieque (LU7); Yunmen (LU2)	ICU mechanical ventilation time, length of stay in the ICU, total blood transfusion, total chest drainage, muscle strength score (1, 6, 12, 24, 48, and 72 h), cTnI (1, 6, 12, 24, 48, and 72 h)	Not mentioned	Shaanxi, China
2	Dai [24]	2019	30	30	36/24	56–80	Percutaneous coronary intervention (PCI)	Electroacupuncture, 6 sessions, 3 days preoperatively and 3 days postoperatively, 1 session per day, 30 min each session	None	Neiguan (PC6); Zusanli (ST36); Guanyuan (RN4)	TCM chest pain symptom score, CK-MB (24 and 48 h), cTnI (24 and 48 h), CRP (24 and 48 h), microalbuminuria creatinine ratio (MACE) status (30 days)	30 days	Sichuan, China
3	Shan et al. [25]	2010	15	15	15/15	30–60	Extracorporeal circulation for the repair of atrial septal defects, ventricular septal defects, mitral valve replacement, and pulmonary sternotomy	Electroacupuncture preconditioning, once, 30 min	None	Neiguan (PC6); Lieque (LU7); Yunmen (LU2)	TNF-α (2 and 24 h), IL-2 (2 and 24 h), IL-10 (2 and 24 h)	Not mentioned	Shanghai, China
4	Zhu et al. [26]	2023	28	29	34/23	18–74	Percutaneous coronary intervention (PCI)	Electroacupuncture preconditioning, once, 30 min	None	Neiguan (PC6); Daling (PC7)	cTnI (24 h), CK (24 h), CK-MB (24 h), toll-like receptor 4 (TLR4) mRNA (24 h), myeloid differentiation factor 88 (MyD88) mRNA (24 h), and nuclear factor-kappa B (NF-κB) mRNA (24 h)	Not mentioned	Shanghai, China
5	Liang et al. [27]	2021	43	43	47/39	18–70	Percutaneous coronary intervention (PCI)	Acupuncture, 14 times, once each day, 30 min each time	None	Neiguan (PC6); Gongsun (SP4); Danzhong (CV17); Xuehai (SP10); Zusanli (ST36); Sanyinjiao (SP6)	CK, AST, LDH, CK-MB, LVEDD, LVESD, LVEF, the Rhode Island methods to improve diagnostic assessment and services (MIDAS) scale, TCM chest pain symptom score, clinical efficacy index	Not mentioned	Beijing, China
6	Xiao et al. [28]	2018	20	20	17/23	18–55	Extracorporeal cardiac surgery	Electroacupuncture preconditioning, 20 min preoperatively to end of the procedure	None	Neiguan (PC6); Ximen (PC4); Shenmen (HT6); Baihui (GV20)	hFABP (0.5, 1, 2, 6, and 24 h), cTnI (0.5, 1, 2, 6, and 24 h) and MDA concentrations (0.5, 1, 2, 6, and 24 h), myocardial contractility score (1, 6, and 24 h), arrhythmia score (24 h)	Not mentioned	Guizhou, China
7	Wang [29]	2019	30	30	40/20	28–64	Heart valve replacement surgery	Electroacupuncture pretreatment, 30 min	Sham electroacupuncture	Baihui (GV20); Yintang (EX-HN3); Shuigou (DU 26)	TNF-α (0, 6, 24, and 72 h), IL-6 (0, 6, 24, and 72 h), IL-10 (0, 6, 24, and 72 h), NSE (0, 6, 24, and 72 h), S100β (0, 6, 24, and 72 h), CAM-ICU scale (3 days), qoR-40 scale (3 days), incidence of POD (3 days), duration of ICU stay, and duration of hospital stay	Not mentioned	Anhui, China
8	Ma et al. [30]	2015	25	25	12/38	36–63	Heart valve replacement surgery	Electroacupuncture preconditioning, 30 min preoperatively to end of the procedure	None	Neiguan (PC6)	MDA (0.5, 6, and 24 h), SOD (0.5, 6, and 24 h), cTnI (0.5, 6, and 24 h), heartbeat recovery rate, dopamine dosage, epinephrine dosage, and nitroglycerin dosage	Not mentioned	Shandong, China
9	Wang et al. [31]	2022	100	100	128/72	65–76	Percutaneous coronary intervention (PCI)	Acupuncture, 14 times, once per day, 30 min	Sham acupuncture	Neiguan (PC6); Shenmen (HT6); Zusanli (ST36)	Incidence of heart failure, IL-1, TNF-α, BNP, LVEF	Not mentioned	Fujian, China
10	Wang et al. [32]	1999	12	16	NA	26–38	Extracorporeal cardiac surgery	Electroacupuncture preconditioning, once, 20–30 min	None	Neiguan (PC6); Lieque (LU7); Yunmen (LU2)	MAP, SpO_2_, dopamine dosage, sodium nitroprusside dosage, complication rate, heartbeat recovery rate	Not mentioned	Shanghai, China
11	Wang et al. [33]	2000	10	9	9/12	26–35	Atrial septal defect repair	Electroacupuncture preconditioning, once, 20–30 min	None	Neiguan (PC6); Lieque (LU7); Yunmen (LU2)	SOD, MDA, CK-MB, mean arterial pressure, heart rate	Not mentioned	Shanghai, China
12	Zhang [34]	2020	30	30	33/27	35–75	Percutaneous coronary intervention (PCI)	Acupuncture, 5 times a week for 1 month	None	Lieque (LU7); hHegu (LI4); Shenmen (HT6); Neiguan (PC6); Quchi (LI11); Chize (LU5)	TCM chest pain symptom score, SF-36 score, hs-CRP, IL-6, TC, TG, LDL-C, ECG changes	Not mentioned	Guangxi, China
13	Zhao et al. [35]	2021	47	47	49/45	65–75	Percutaneous coronary intervention (PCI)	Transcutaneous electrical stimulation of acupuncture points, 30 min preoperatively to the end of the procedure	Sham transcutaneous electrical stimulation at acupuncture points	Neiguan (PC); ximen (PC4)	ET-1 (8 and 21 h), vWF (8 and 21 h), NO (8 and 21 h), FMD (8 and 21 h), IL-6 (8 and 21 h), IL-10 (8 and 21 h), MMP-9 (8 and 21 h), hs-CRP (8 and 21 h)	Not mentioned	Hebei, China
14	Chi et al. [36]	2014	80	80	70/90	51–71	Heart valve replacement surgery	Electroacupuncture preconditioning, once, 20–30 min	None	Zhongfu (LU1); Chize (LU5); Ximen (PC4)	Surgical success rate, anesthetic drug dosage, duration of surgery, duration of aortic block, heartbeat recovery rate, postoperative blood loss, drainage volume, pulmonary infection, vocal cord injury, time to first mobilization from bed, time to first postoperative meal, duration of ICU stay, duration of antibiotic use, post-operative hospital stay duration, total healthcare expenses	Not mentioned	Shanghai, China
15	Wang et al. [37]	2015	102	102	148/56	≥ 18	Percutaneous coronary intervention (PCI)	Electroacupuncture pretreatment, once, 30 min	Sham electroacupuncture	Neiguan (PC6); Ximen (PC4)	cTnI (24 h), MI4a incidence (24 h), LVEDD (0, 3, and 6 m), EDV, SV, LVEF, MACE rate (24 m), hs-CRP (24 h), TNF-α (24 h), IL-6 (24 h), IL-10 (24 h), HMGB1 (24 h), PET/CT imaging myocardial metabolic activity	24 months	Shaanxi, China
16	Ni et al. [21]	2012	34	36	34/36	2–12	Pediatric congenital heart disease	Transcutaneous electrical stimulation of acupuncture points, 30 min preoperatively	Sham transcutaneous electrical stimulation at acupuncture points	Neiguan (PC6)	cTnI (0.5, 2, 8, and 24 h), hs-CRP (0.5, 2, 8, and 24 h), IL-6 (0.5, 2, 8, and 24 h), IL-10 (0.5, 2, 8, and 24 h), TNF-a (0.5, 2, 8, and 24 h), mechanical ventilation time, urinary output, rate of in-hospital reoperation, postoperative complications, length of stay in the ICU, duration of hospital stay, in-hospital mortality rate, muscle strength score	Not mentioned	Shaanxi, China
17	Pei et al. [38]	2024	70	70	93/47	53–80	Percutaneous coronary intervention (PCI)	Electroacupuncture was carried out for 30 min each time, once a day, for a total of 3 days	Sham electroacupuncture	Neiguan (PC6); Daling (PC7); Xiajuxu point (ST39)	Fasting blood glucose, fasting insulin, HOMA-IR, IL-6, Hs-CRP, AoPPs, OX-LDL, Hamilton Anxiety Scale (HAMA), Pittsburgh Sleep Quality Index (PSQI), heart rate variability	3 days	Beijing, China
18	Mu et al. [39]	2024	47	47	56/38	42–53	Heart bypass surgery	Electroacupuncture pretreatment was conducted for 30 min each day and continued for 5 consecutive days	None	Neiguan (PC6); Lieque (LU7); Yunmen (LU2)	cTnI (12 and 24 h), hs-CRP (12 and 24 h), LVEF (5 days), E/A (5 days), the incidence of postoperative adverse cardiac events	Not mentioned	Hebei, China
19	Wang [40]	2016	38	38	47/29	45–77	Percutaneous coronary intervention (PCI)	Acupuncture was performed five times a week, with each session lasting 25 min	None	Neiguan (PC6); Shenmen (HT6); Xinshu (BL15); Daling (PC7); Sanyinjiao (SP6)	TG (6 weeks), TC (6 weeks), LDH (6 weeks), 6 min walk test (6MWT) (6 weeks), LEDV (6 weeks), Seattle Angina Questionnaire (SAQ) scale (6 weeks)	Not mentioned	Hubei, China
20	Zhang [41]	2022	42	45	53/34	47–76	Percutaneous coronary intervention (PCI)	Acupuncture and moxibustion were carried out once a week for 30 min each time, starting before reperfusion treatment and continuing until the seventh day after the operation	None	Neiguan (PC6)	CK-MB (6, 12, 24, 48, and 72 h and 7 days), MYO (6, 12, 24, 48, and 72 h and 7 days), cardiac arrhythmia (total premature ventricular contractions, single premature ventricular contractions, runs of premature ventricular contractions, ventricular tachycardia), NT-proBNP level (24 and 72 h and 7 days), EDV (7 days), ESV (7* *days), IVS (7 days), IVPW (7 days), LVE (7 days)	Not mentioned	Hubei, China
21	Ping et al. [42]	2003	24	24	None	Not mentioned	Coronary artery bypass grafting	Electroacupuncture pretreatment was carried out once, lasting for 20–30 min	None	Neiguan(PC6); Lieque (LU7); Yunmen (LU2)	IL-8, SOD, MDA, ultrastructural changes of cardiomyocytes	Not mentioned	Shanghai, China
22	Si [43]	2020	19	18	25/12	35–85	Percutaneous coronary intervention (PCI)	Transcutaneous electrical acupoint stimulation was given for 30 min each time, once a day, for a total of 3 days of treatment	None	Neiguan (PC6); Daling (PC7); Jueyinshu (BL14); Xinshu (BL15)	TCM clinical symptom score (24 h), overall efficacy evaluation, self-rating anxiety scale (SAS) score (24 h), self-rating depression scale (SDS) score (24 h), CK, CK-MB, LDH, MYO	Not mentioned	Hunan, China
23	He et al. [44]	2024	38	38	35/41	40–68	Percutaneous coronary intervention (PCI)	Auricular point pressing with beans was performed twice a day for 4 weeks	None	Shenmen (TF4), sympathetic (AH6a), subcortex, heart (CO15), spleen (CO13), stomach (CO13)	Hamilton Anxiety Scale (HAMA), Hamilton Depression Scale (HAMD), PSQI, 6 min walk test (6MWT), LVEDD, LVESD, LVEF, CI, SAQ, World Health Organization quality of life–brief (WHOQOL–BREF), coincidence of adverse cardiovascular events (myocardial infarction, angina pectoris, cardiac arrhythmia)	6 months	Jiangxi, China
24	Jia et al. [45]	2022	15	15	17/13	45–71	Heart valve replacement surgery	Transcutaneous electrical acupoint stimulation, starting 30 min before the operation and lasting until the operation is completed	Sham transcutaneous electrical acupoint stimulation	Shenting (DU24), Dazhui (DU14)	The incidence of POD (1, 2, and 3 days), cardiopulmonary bypass time, duration of aortic block, operation time, total fluid output, total fluid intake, sufentanil dosage, propofol dosage, sevoflurane dosage, duration of tube-indwelling, duration of ICU stay, duration of hospital stay, fentanyl dosage, midazolam dosage, IL-6 (1 and 3 days)	Not mentioned	Guangdong, China
25	Wang et al. [46]	2012	20	20	21/19	30–60	Mitral valve replacement	Electroacupuncture induction was carried out from 1 h before the operation until the operation was completed	None	Neiguan (PC6); Yunmen (LU2); Lieque (LU7)	RBC-C3b RR (0.5 and 24 h), RBC-ICR(0.5 and 24 h), MDA(0.5 and 24 h), SOD(0.5 and 24 h)	Not mentioned	Guizhou, China
26	Shao et al. [47]	2024	21	21	30/12	44–68	Percutaneous coronary intervention (PCI)	Acupuncture was administered three times a week, with each session lasting 30 min and a treatment course of 8 weeks	None	Jiaji (EX-B2)	6-min walk test (6MWT) (8w), LVEF (8 weeks), grip strength of a single hand (8 weeks), the number of times of voluntarily lifting a unilateral lower limb within 10 s (8 weeks), cardiac arrhythmia status (8 weeks), International Classification of Functioning (ICF) Score (8 weeks)	Not mentioned	Shanghai, China

**Table 3:** Comparison of cTnI levels as markers of myocardial injury in the acupuncture and the control groups.

Indicators	Acupuncture group (*x* ± *s*, *n*)	Control group (*x* ± *s*, *n*)	SMD random (95% CI)	*I* ^2^ (%)	*p*	*Z*	*p*
*cTnI (0.25 h)*							
Ma et al. 2015	0.27 ± 0.08, 25	0.26 ± 0.47, 25	0.03 [−0.53, 0.58]			0.10	0.92

*cTnI (0.5 h)*							
Ma et al. 2015	3.07 ± 0.63, 25	4.67 ± 0.51, 25					
Xiao et al. 2018	0.226 ± 0.021, 20	0.275 ± 0.019, 20					
Ni et al. 2012	6.96 ± 2.13, 34	9.47 ± 1.93, 36					
	*n* = 79	*n* = 81	−2.08 [−3.09, −1.07]	84	0.002	4.04	< 0.0001

*cTnI (1 h)*							
Xiao et al. 2018	0.224 ± 0.025, 20	0.256 ± 0.024, 20					
Yang et al. 2010	0.14 ± 0.39, 30	0.15 ± 0.37, 30					
	*n* = 50	*n* = 50	−0.63 [−1.86, 0.60]	88	0.004	1.01	0.31

*cTnI (2 h)*							
Xiao et al. 2018	0.212 ± 0.022, 20	0.259 ± 0.021, 20					
Ni et al. 2012	11.21 ± 2.03, 34	14.69 ± 1.93, 36					
	*n* = 54	*n* = 56	−1.87 [−2.33, −1.42]	0	0.41	8.07	< 0.00001

*cTnI (3 h)*							
Yang et al. 2010	3.44 ± 3.68, 30	5.51 ± 3.67, 30	−0.56 [−1.07, −0.04]			2.11	0.03

*cTnI (6 h)*							
Ma et al. 2015	4.08 ± 0.94, 25	5.62 ± 1.09, 25					
Xiao et al. 2018	0.197 ± 0.21, 20	0.246 ± 0.025, 20					
Yang et al. 2010	5.74 ± 3.62, 30	7.89 ± 4.04, 30					
	*n* = 75	*n* = 75	−0.78 [−1.44, −0.11]	74	0.02	2.30	0.02

*cTnI (8 h)*							
Ni et al. 2012	9.18 ± 1.74, 34	13.24 ± 1.93, 36	−2.18 [−2.78, −1.58]			7.15	< 0.00001

*cTnI (12 h)*							
Yang et al. 2010	6.22 ± 3.53, 30	8.34 ± 5.93, 30					
Mu et al. 2024	0.65 ± 0.1, 47	0.91 ± 0.13, 47					
	*n* = 77	*n* = 77	−1.33 [−3.08, 0.43]	96	< 0.00001	1.48	0.14

*cTnI (24 h)*							
Ma et al. 2015	2.48 ± 0.75, 25	3.69 ± 0.89, 25					
Xiao et al. 2018	0.171 ± 0.016, 20	0.221 ± 0.018, 20					
Yang et al. 2010	6.22 ± 3.53, 30	8.34 ± 5.93, 30					
Ni et al. 2012	6.14 ± 1.01, 34	8.5 ± 1.11, 36					
Mu et al. 2024	0.41 ± 0.08, 47	0.67 ± 0.11, 47					
Zhu et al. 2023	0.008 ± 0.1393, 28	0.04 ± 0.1209, 29					
Dai 2019	0.0727 ± 0.03118, 30	0.0957 ± 0.04042, 30					
	*n* = 214	*n* = 217	−1.47 [−2.27,−0.68]	92	< 0.00001	3.65	0.0003

*cTnI (48 h)*							
Yang et al. 2010	3.97 ± 3.83, 30	5.43 ± 7.6, 30					
Dai 2019	0.0923 ± 0.03081, 30	0.126 ± 0.04031, 30					
	*n* = 60	*n* = 60	−0.58 [−1.25, 0.10]	70	0.07	1.68	0.09

*cTnI (72 h)*							
Yang et al. 2010	1.97 ± 2.07, 30	2.65 ± 2.45, 30	−0.30 [−0.80, 0.21]			1.14	0.25
Total	*n* = 728	*n* = 737	−1.22 [−1.57,−0.87]	89	< 0.00001	6.77	< 0.00001

**Table 4:** Comparison of CK-MB levels as myocardial injury markers in the acupuncture and control groups.

Indicators	Acupuncture group (*x* ± *s*, *n*)	Control group (*x* ± *s*, *n*)	SMD fixed (95% CI)	*I* ^2^ (%)	*p*	*Z*	*p*
*CK-MB (6 h)*							
Zhang 2022	389.35 ± 12.7, 42	453.96 ± 19.54, 45	−3.86 [−4.58,−3.14]			10.48	< 0.00001

*CK-MB (12 h)*							
Zhang 2022	204.49 ± 16.66, 42	376.21 ± 39.06, 45	−5.60 [−6.55,−4.65]			11.56	< 0.00001

*CK-MB (24 h)*							
Zhang 2022	126.57 ± 2.67, 42	228.53 ± 7.66, 45					
Zhu et al. 2023	1.2 ± 1.19, 30	1.5 ± 2.56, 30					
Dai 2019	46.43 ± 8.02, 28	53.03 ± 7.81, 29					
	*n* = 100	*n* = 104	−0.78 [−1.15,−0.41]	99	< 0.00001	4.17	< 0.0001

*CK-MB (48 h)*							
Zhang 2022	51.1 ± 8.13, 43	50.89 ± 5.57, 45					
Dai 2019	47.63 ± 8.02, 30	54.93 ± 7.53, 30					
	*n* = 73	*n* = 75	−0.33 [−0.66,−0.00]	87	0.006	1.98	0.05

*CK-MB (72 h)*							
Fu et al. 2020	12.08 ± 3.81, 19	11.09 ± 5.03, 18					
Zhang 2022	20.99 ± 4.83, 42	21.31 ± 3.47, 45					
	*n* = 61	*n* = 63	0.01 [−0.34, 0.36]	0	0.46	0.06	0.95

*CK-MB (7 days)*							
Zhang 2022	6.1 ± 0.96, 42	6.34 ± 1.69, 45	−0.17 [−0.59, 0.25]			0.80	0.42

*CK-MB (14 days)*							
Liang et al. 2021	25.16 ± 8.14, 43	31.54 ± 8.35, 43	−0.77 [−1.21,−0.33]			3.43	0.0006
Total	*n* = 403	*n* = 420	−0.71 [−0.87,−0.55]	97	< 0.00001	8.63	< 0.00001

**Table 5:** Comparison of BNP levels as myocardial injury markers in the acupuncture and control groups.

Indicators	Acupuncture group (*x* ± *s*, *n*)	Control group (*x* ± *s*, *n*)	SMD random (95% CI)	*I* ^2^ (%)	*p*	*Z*	*p*
*BNP(1d)*							
Zhang 2022	972.21 ± 120.24, 42	1746.51 ± 230.64, 45	−4.13 [−4.89,−3.38]			10.71	< 0.00001

*BNP(3d)*							
Zhang 2022	529.24 ± 84.18, 42	1034.27 ± 170.04, 45	−3.69 [−4.39,−2.99]			10.32	< 0.00001

*BNP(7d)*							
Zhang 2022	227.05 ± 48.28, 42	596.11 ± 147.3, 45	−3.29 [−3.94,−2.64]			9.87	< 0.00001

*BNP(14d)*							
Wang et al. 2022	3.35 ± 1.01, 100	3.87 ± 1.08, 100	−0.50 [−0.78,−0.21]			3.45	0.0006
Total	*n* = 226	*n* = 235	−2.89 [−4.95,−0.83]	98	< 0.00001	2.75	0.006

**Table 6:** Comparison of hs-CRP levels (an inflammatory cytokine) in the acupuncture and control groups.

Indicators	Acupuncture group (*x* ± *s*, *n*)	Control group (*x* ± *s*, *n*)	SMD random (95% CI)	*I* ^2^ (%)	*p*	*Z*	*p*
*hs-CRP(0.5 h)*							
Ni et al. 2012	608 ± 74.67, 34	736 ± 149.33, 36	−1.06 [−1.57,−0.56]			4.15	< 0.0001

*hs-CRP(2 h)*							
Ni et al. 2012	736 ± 117.33, 34	992 ± 160, 36	−1.80 [−2.36,−1.24]			6.29	< 0.00001

*hs-CRP(6 h)*							
Zhang 2020	12.8 ± 2.98, 30	12.23 ± 2.43, 30	0.21 [−0.30, 0.71]			0.80	0.42

*hs-CRP(8 h)*							
Zhao et al. 2021	12.51 ± 1.5, 47	16.81 ± 1.64, 47					
Ni et al. 2012	1525.33 ± 181.33, 34	2133.33 ± 170.67, 36					
	*n* = 81	*n* = 83	−3.02 [−3.71,−2.34]	54	0.14	8.65	< 0.00001

*hs-CRP(12 h)*							
Mu et al. 2024	60.22 ± 5.03, 47	68.74 ± 5.37, 47	−1.62 [−2.09,−1.16]			6.79	< 0.00001

*hs-CRP(24 h)*							
Zhao et al. 2021	8.43 ± 1.45, 47	13.63 ± 1.64, 47					
Wang et al. 2015	1807.14 ± 178.57, 102	1914.29 ± 200, 102					
Ni et al. 2012	2976 ± 149.33, 34	3242.67 ± 138.67, 36					
Mu et al. 2024	43.26 ± 4.97, 47	50.14 ± 5.63, 47					
Dai 2019	8.13 ± 0.724, 30	8.53 ± 0.867, 30					
	*n* = 260	*n* = 262	−1.48 [−2.36,−0.59]	95	< 0.00001	3.27	0.001

*hs-CRP(48 h)*							
Dai 2019	4.17 ± 0.685, 30	4.57 ± 0.908, 30	−0.49 [−1.01, 0.02]			1.87	0.06

*hs-CRP(72 h)*							
Pei 2024	−0.01 ± 0.01, 70	−0.01 ± 0.03, 70	0.00 [−0.33, 0.33]			0.00	1.00

*hs-CRP(30d)*							
Zhang 2020	7.19 ± 1.52, 30	8.13 ± 1.33, 30	−0.65 [−1.17,−0.13]			2.45	0.01
Total	*n* = 616	*n* = 624	−1.34 [−1.87,−0.80]	94	< 0.00001	4.92	< 0.00001

**Table 7:** Inflammatory cytokine, TNF-α levels in the acupuncture and control groups.

Indicators	Acupuncture group (*x* ± *s*, *n*)	Control group (*x* ± *s*, *n*)	SMD random (95% CI)	*I* ^2^ (%)	*p*	*Z*	*p*
*TNF-α (0 h)*							
Wang et al. 2019	16.3 ± 2.6, 30	21.2 ± 3.7, 30	−1.51 [−2.09,−0.93]			5.13	< 0.00001

*TNF-α (0.5 h)*							
Ni et al. 2012	63.13 ± 6.57, 34	79.29 ± 10.61, 36	−1.80 [−2.36,−1.24]			6.30	< 0.00001

*TNF-α (2 h)*							
Shan et al. 2010	98.67 ± 35.12, 15	123.1 ± 47.21, 15					
Ni et al. 2012	97.98 ± 14.14, 34	104.04 ± 21.72, 36					
	*n* = 49	*n* = 51	−0.40 [−0.79,−0.00]	0	0.58	1.96	0.05

*TNF-α (6 h)*							
Wang et al. 2019	32.1 ± 5.1, 30	38.7 ± 5.6, 30	−1.22 [−1.77,−0.66]			4.30	< 0.0001

*TNF-α (8 h)*							
Ni et al. 2012	102.02 ± 16.16, 34	109.09 ± 18.69, 36	−0.40 [−0.87, 0.07]			1.65	0.10

*TNF-α (24 h)*							
Shan et al. 2010	35.17 ± 10.56, 15	49.01 ± 13.75, 15					
Wang et al. 2015	111.9 ± 13.1, 102	136.9 ± 20.24, 102					
Ni et al. 2012	138.38 ± 23.74, 34	156.57 ± 25.25, 36					
Wang et al. 2019	41.5 ± 6.9, 30	52.6 ± 8.7, 30					
	*n* = 181	*n* = 183	−1.20 [−1.57,−0.83]	54	0.09	6.34	< 0.00001

*TNF-α (72 h)*							
Wang et al. 2019	38.2 ± 6.4, 30	49.7 ± 8.3, 30	−1.53 [−2.11,−0.95]			5.18	< 0.00001

*TNF-α (14 days)*							
Wang et al. 2022	30.66 ± 4.04, 100	36.59 ± 4.21, 100	−1.43 [−1.74,−1.12]			9.01	< 0.00001
Total	*n* = 488	*n* = 496	−1.13 [−1.41,−0.85]	74	< 0.0001	7.84	< 0.00001

**Table 8:** Inflammatory cytokine, IL-10 levels in the acupuncture and control groups.

Indicators	Acupuncture group (*x* ± *s*, *n*)	Control group (*x* ± *s*, *n*)	SMD random (95% CI)	*I* ^2^ (%)	*p*	*Z*	*p*
*IL-10 (0 h)*							
Wang et al. 2019	76 ± 7, 30	68 ± 7, 30	1.13 [0.58, 1.68]			4.044	< 0.0001

*IL-10 (0.5 h)*							
Ni et al. 2012	87.5 ± 8.75, 34	98.27 ± 8.75, 36	−1.22 [−1.73,−0.70]			4.65	< 0.00001

*IL-10 (2 h)*							
Shan et al. 2010	595.8 ± 46.7, 15	381.6 ± 23.5, 15					
Ni et al. 2012	107.36 ± 5.72, 34	103.65 ± 12.79, 36					
	*n* = 49	*n* = 51	2.94 [−2.23, 8.10]	97	< 0.00001	1.11	0.26

*IL-10 (6 h)*							
Wang et al. 2019	92 ± 11, 30	77 ± 8, 30	1.54 [0.96, 2.12]			5.20	< 0.00001

*IL-10 (8 h)*							
Zhao et al. 2021	337.1 ± 53.17, 47	260.18 ± 42.99, 47					
Ni et al. 2012	98.27 ± 10.1, 34	91.2 ± 9.76, 36					
	*n* = 81	*n* = 83	1.14 [0.29, 2.00]	85	0.01	2.62	0.009

*IL-10 (24 h)*							
Zhao et al. 2021	226.24 ± 40.72, 47	154.98 ± 35.07, 47					
Shan et al. 2010	182.9 ± 25.2, 15	141.5 ± 21.8, 15					
Wang et al. 2015	222.69 ± 36.13, 102	148.74 ± 30.25, 102					
Ni et al. 2012	92.88 ± 7.4, 34	84.81 ± 7.4, 36					
Wang et al. 2019	107 ± 13, 30	96 ± 12, 30					
	*n* = 228	*n* = 230	1.55 [1.00, 2.11]	83	< 0.0001	5.47	< 0.00001

*IL-10 (72 h)*							
Wang et al. 2019	94 ± 10, 30	82 ± 9, 30	1.25 [0.69, 1.80]			4.39	< 0.00001
Total	*n* = 482	*n* = 490	1.31 [0.74, 1.87]	93	< 0.00001	4.55	< 0.00001

**Table 9:** Inflammatory cytokine, IL-6 levels in the acupuncture and control groups.

Indicators	Acupuncture group (*x* ± *s*, *n*)	Control group (*x* ± *s*, *n*)	SMD random (95% CI)	*I* ^2^ (%)	*p*	*Z*	*p*
*IL-6 (0 h)*							
Wang et al. 2019	27.6 ± 4.6, 30	35.4 ± 5.3, 30	−1.55 [−2.13, −0.97]			5.23	< 0.00001

*IL-6 (0.5 h)*							
Ni et al. 2012	208.69 ± 23.4, 34	247.06 ± 29.95, 36	−1.41 [−1.93, −0.88]			5.24	< 0.00001

*IL-6 (2 h)*							
Ni et al. 2012	239.57 ± 24.33, 34	267.65 ± 29.95, 36	−1.01 [−1.51, −0.52]			3.98	< 0.0001

*IL-6 (6 h)*							
Wang et al. 2019	41.2 ± 5.4, 30	49.4 ± 6.1, 30					
Zhang 2020	65.47 ± 9.57, 30	65.43 ± 10.3, 30					
	*n* = 60	*n* = 60	−0.69 [−2.07, 0.69]	92	0.0003	0.99	0.32

*IL-6 (8 h)*							
Zhao et al. 2021	187.74 ± 32.26, 47	230.32 ± 40.65, 47					
Ni et al. 2012	196.52 ± 23.4, 34	235.83 ± 28.07, 36					
	*n* = 81	*n* = 83	−1.29 [−1.63, −0.95]	0	0.32	7.48	< 0.00001

*IL-6 (24 h)*							
Jia et al. 2022	12.72 ± 3.51, 15	18.95 ± 1.89, 15					
Zhao et al. 2021	116.13 ± 38.06, 47	208.39 ± 42.58, 47					
Wang et al. 2015	125.7 ± 21.79, 102	211.17 ± 41. 06, 102					
Ni et al. 2012	186.23 ± 27.14, 34	217.11 ± 37.43, 36					
Wang et al. 2019	39.2 ± 5.5, 30	46.1 ± 5.7, 30					
	*n* = 228	*n* = 230	−1.83 [−2.54, −1.11]	89	< 0.00001	5.02	< 0.00001

*IL-6 (72 h)*							
Jia et al. 2022	5.7 ± 1.04, 15	7.88 ± 2.49, 15					
Pei et al. 2024	−0.008 ± 0.009, 70	−0.006 ± 0.007, 70					
Wang et al. 2019	15.7 ± 3.1, 30	46.1 ± 5.7, 30	−6.54 [−7.85, −5.23]			9.77	< 0.00001
	*n* = 115	*n* = 115	−2.55 [−5.30, 0.20]	98	< 0.00001	1.82	0.07

*IL-6 (30 days)*							
Zhang 2020	16.41 ± 5.95, 30	21.3 ± 3.4, 30	−1.00 [−1.53, −0.46]			3.62	0.0003
Total	*n* = 612	*n* = 620	−1.53 [−2.01, −1.06]	92	< 0.00001	6.33	< 0.00001

**Table 10:** The levels of oxidative stress factor MDA in the acupuncture and control groups.

Index	Acupuncture group (*x* ± *s*, *n*)	Control group (*x* ± *s*, *n*)	SMD random (95% CI)	*I* ^2^ (%)	*p*	*Z*	*p*
*MDA (0 h)*							
Wang et al. 2000	4.51 ± 1.58, 10	4.81 ± 0.99, 9					
Wang et al. 2012	8.3 ± 1, 20	10.9 ± 1.4, 20					
Lu et al. 2003	4.9 ± 0.4, 24	5.6 ± 0.2, 24					
	*n* = 54	*n* = 53	−1.52 [−2.70,−0.34]	85	0.002	2.53	0.01

*MDA (0.25 h)*							
Ma et al. 2015	4.57 ± 1.87, 25	4.41 ± 2.01, 25	0.08 [−0.47, 0.64]			0.29	0.77

*MDA (0.5 h)*							
Ma et al. 2015	8.47 ± 2.64, 25	11.84 ± 3.02, 25					
Xiao et al. 2018	5.8 ± 2.1, 20	8.6 ± 2.8, 20					
	*n* = 45	*n* = 45	−1.14 [−1.59, −0.69]	0	0.90	4.99	< 0.00001

*MDA (1 h)*							
Xiao et al. 2018	5.6 ± 0.5, 20	8.2 ± 0.6, 20					
Wang et al. 2000	4.67 ± 2.38, 10	7.99 ± 1.71, 9					
	*n* = 30	*n* = 29	−3.05 [−6.08, −0.01]	93	0.0002	1.97	0.05

*MDA (2 h)*							
Xiao et al. 2018	6 ± 0.4, 20	7.3 ± 0.5, 20	−2.81 [−3.71, −1.92]			6.14	< 0.00001

*MDA (6 h)*							
Ma et al. 2015	11.02 ± 2.65, 25	15.11 ± 2.83, 25					
Xiao et al. 2018	5.7 ± 0.4, 20	6.9 ± 0.4, 20					
	*n* = 45	*n* = 45	−2.16 [−3.61, −0.72]	85	0.010	2.95	0.003

*MDA (24 h)*							
Ma et al. 2015	8.37 ± 1.98, 25	12.41 ± 2.31, 25					
Xiao et al. 2018	4.6 ± 0.3, 20	5.2 ± 0.3, 20					
	*n* = 45	*n* = 45	−1.90 [−2.40, −1.39]	0	0.83	7.36	< 0.00001
Total	*n* = 264	*n* = 262	−1.78 [−2.36, −1.20]	87	< 0.00001	6.00	< 0.00001

**Table 11:** Levels of oxidative stress factor SOD in acupuncture and control groups.

Index	Acupuncture group (*x* ± *s*, *n*)	Control group (*x* ± *s*, *n*)	SMD random (95% CI)	*I* ^2^ (%)	*p*	*Z*	*p*
*SOD (0 h)*							
Wang et al. 2000	89.9 ± 14.74, 10	69.14 ± 9.49, 9					
Wang et al. 2012	80 ± 11, 20	71 ± 12, 20					
Lu et al. 2023	19.2 ± 2.8, 24	16.6 ± 5, 24					
	*n* = 54	*n* = 53	0.84 [0.39, 1.28]	17	0.30	3.67	0.0002

*SOD (0.25 h)*							
Ma et al. 2015	30.2 ± 4.37, 25	31.18 ± 5.43, 25	−0.20 [−0.75, 0.36]			0.69	0.49

*SOD (0.5 h)*							
Ma et al. 2015	24.8 ± 5.14, 25	17.03 ± 5.06, 25	1.50 [0.87, 2.13]			4.64	< 0.00001

*SOD (1 h)*							
Wang et al. 2000	102.09 ± 16.59, 10	68 ± 24.77, 9	1.56 [0.50, 2.62]			2.89	0.004

*SOD (6 h)*							
Ma et al. 2015	21.5 ± 3.67, 25	16.1 ± 3.25, 25	1.53 [0.90, 2.17]			4.72	< 0.00001

*SOD (24 h)*							
Ma et al. 2015	24.57 ± 5.04, 25	18.42 ± 4.35, 25				4.11	< 0.0001
Wang et al. 2012	85 ± 12, 20	77 ± 13, 20					
	*n* = 45	*n* = 45	0.96 [0.32, 1.61]	53	0.14	2.92	0.004
Total	*n* = 184	*n* = 182	0.98 [0.55, 1.41]	72	0.0004	4.50	< 0.00001

**Table 12:** LVEF levels in cardiac ultrasound function index for acupuncture and control groups.

Index	Acupuncture group (*x* ± *s*, *n*)	Control group (*x* ± *s*, *n*)	SMD random (95% CI)	*I* ^2^ (%)	*p*	*Z*	*p*
*LVEF (0 days)*							
Wang et al. 2015	54.56 ± 0.77, 102	52.81 ± 0.97, 102	1.99 [1.65, 2.33]			11.59	< 0.00001

*LVEF (5 days)*							
Mu et al. 2024	57.14 ± 3.97, 47	56.76 ± 3.58, 47	0.10 [−0.30, 0.50]			0.48	0.63

*LVEF (7 days)*							
Zhang et al. 2022	52.6 ± 9.04, 42	46.96 ± 8.29, 45	0.65 [0.21, 1.08]			2.93	0.003

*LVEF (14 days)*							
Liang et al. 2021	59.62 ± 5.41, 43	55.82 ± 5.75, 43					
Wang et al. 2022	52.65 ± 8.65, 100	50.545 ± 8.46, 100					
	*n* = 143	*n* = 143	0.43 [0.01, 0.84]	62	0.10	2.01	0.04

*LVEF (28 days)*							
He et al. 2024	48.34 ± 5.31, 38	40.62 ± 5.19, 38	1.46 [0.95, 1.96]			5.61	< 0.00001

*LVEF (42 days)*							
Wang 2016	58 ± 7.1, 38	54 ± 6.7, 38	0.57 [0.11, 1.03]			2.45	0.01

*LVEF (56 days)*							
Shao et al. 2024	57.33 ± 4.27, 21	54.52 ± 4.29, 21	0.64 [0.02, 1.27]			2.03	0.04

*LVEF (90 days)*							
Wang et al. 2015	55.96 ± 0.81, 102	52.43 ± 1.41, 102	3.06 [2.65, 3.46]			14.75	< 0.00001

*LVEF (180 days)*							
Wang et al. 2015	57.15 ± 0.5, 102	54.9 ± 0.67, 102	3.79 [3.33, 4.25]			16.09	< 0.00001
Total	*n* = 635	*n* = 638	1.32 [0.54, 2.09]	97	< 0.00001	3.33	0.0009

**Table 13:** LVEDD levels in cardiac ultrasound function index for acupuncture and control groups.

Indicators	Acupuncture group (*x* ± *s*, *n*)	Control group (*x* ± *s*, *n*)	SMD random (95% CI)	*I* ^2^ (%)	*p*	*Z*	*p*
*LVEDD (0 h)*							
Wang et al. 2015	49.74 ± 0.77, 102	49.38 ± 0.67, 102	0.50 [0.22, 0.78]			3.49	0.0005

*LVEDD (14 days)*							
Liang et al. 2021	48.55 ± 2.76, 43	50.43 ± 2.41, 43	−0.72 [−1.16, −0.28]			3.23	0.001

*LVEDD (28 days)*							
He et al. 2024	56.31 ± 4.84, 38	61.44 ± 5.21, 38	−1.01 [−1.49, −0.53]			4.13	< 0.0001

*LVEDD (90 days)*							
Wang et al. 2015	50.68 ± 1.12, 102	48.7 ± 0.79, 102	2.04 [1.70, 2.37]			11.76	< 0.00001

*LVEDD (180 days)*							
Wang et al. 2015	43.56 ± 1.1, 102	47.28 ± 0.93, 102	−3.64 [−4.09, −3.19]			15.85	< 0.00001
Total	*n* = 387	*n* = 387	−0.56 [−2.33, −1.21]	99	< 0.00001	0.62	0.53

**Table 14:** LVEDV levels in cardiac ultrasound function index for acupuncture and control groups.

Indicators	Acupuncture group (*x* ± *s*, *n*)	Control group (*x* ± *s*, *n*)	SMD random (95% CI)	*I* ^2^ (%)	*p*	*Z*	*p*
*LVEDV (0 days)*							
Wang et al. 2015	99.51 ± 3.82, 102	96.48 ± 3.24, 102	0.85 [0.57, 1.14]			5.82	< 0.00001

*LVEDV (7 days)*							
Zhang 2022	104 ± 12.18, 42	103.22 ± 12.84, 45	0.06 [−0.36, 0.48]			0.29	0.77

*LVEDV (90 days)*							
Wang et al. 2015	92.64 ± 6.19, 102	92.96 ± 5.88, 102	−0.55 [−0.33, 0.22]			0.38	0.71

*LVEDV (180 days)*							
Wang et al. 2015	83.47 ± 3.23, 102	96.24 ± 5.41, 102	−2.86 [−3.25. −2.46]			14.28	< 0.00001
Total	*n* = 348	*n* = 351	−0.50 [−1.96, 0.97]	99	< 0.00001	0.66	0.51

**Table 15:** LVESV levels in cardiac ultrasound function index for acupuncture and control groups.

Indicators	Acupuncture group (*x* ± *s*, *n*)	Control group (*x* ± *s*, *n*)	SMD random (95% CI)	*I* ^2^ (%)	*p*	*Z*	*p*
*LVESV (0 days)*							
Wang et al. 2015	55.63 ± 1.67, 102	53.85 ± 1.56, 102	1.10 [0.80, 1.39]			7.30	< 0.00001

*LVESV (7 days)*							
Zhang 2022	31.31 ± 4.95, 42	29.53 ± 6.31, 45	0.31 [−0.11, 0.73]			1.43	0.15

*LVESV (90 days)*							
Wang et al. 2015	54.5 ± 3.08, 102	53.85 ± 2.78, 102	0.22 [−0.05, 0.50]			1.57	0.12

*LVESV (180 days)*							
Wang et al. 2015	65.68 ± 2.17, 102	58.68 ± 1.72, 102	3.56 [3.12, 4.01]			15.72	< 0.00001
Total	*n* = 348	*n* = 351	1.29 [−0.01, 2.59]	98	< 0.00001	1.95	0.05

**Table 16:** Assessment of evidence quality using the GRADE framework.

Outcome measures	Number of participants (number of studies)	Limitations	Inconsistency	Indirectness	Inaccuracy	Publication bias	Quality of evidence
cTnI	1465 (8)	−1^a^	−2^c^	0	0	0	⊕⊝⊝⊝ Very low quality
CK-MB	823 (5)	−1^a^	−2^c^	0	0	0	⊕⊝⊝⊝ Very low quality
CK	180 (3)	−1^a^	−2^c^	0	−1^d^	0	⊕⊝⊝⊝ Very low quality
AST	86 (1)	−1^a^	0	0	−1^d^	0	⊕⊕⊝⊝ Low quality
LDH	123 (2)	−1^a^	−2^c^	0	−2^e^	0	⊕⊝⊝⊝ Very low quality
BNP	461 (2)	−1^a^	−2^c^	0	0	0	⊕⊝⊝⊝ Very low quality
hs-CRP	1240 (7)	−1^a^	−2^c^	0	0	0	⊕⊝⊝⊝ Very low quality
TNF-α	364 (4)	−1^a^	−2^c^	0	−1^d^	0	⊕⊝⊝⊝ Very low quality
IL-10	458 (5)	−1^a^	−2^c^	0	0	0	⊕⊝⊝⊝ Very low quality
IL-6	1232 (7)	−1^a^	−2^c^	0	0	0	⊕⊝⊝⊝ Very low quality
IL-2	60 (1)	−1^a^	0	0	−1^d^	0	⊕⊕⊝⊝ Low quality
IL-1	200 (1)	−1^a^	0	0	−1^d^	0	⊕⊕⊝⊝ Low quality
IL-8	48 (1)	−1^a^	0	0	−1^d^	0	⊕⊕⊝⊝ Low quality
MDA	526 (5)	−1^a^	−2^c^	0	0	0	⊕⊝⊝⊝ Very low quality
SOD	366 (4)	−1^a^	−1^b^	0	−1^d^	0	⊕⊝⊝⊝ Very low quality
LVEF	1273 (8)	−1^a^	−2^c^	0	0	0	⊕⊝⊝⊝ Very low quality
LVFS	612 (1)	−1^a^	−2^c^	0	0	0	⊕⊝⊝⊝ Very low quality
LVEDD	774 (3)	−1^a^	−2^c^	0	−1^d^	0	⊕⊝⊝⊝ Very low quality
LVEDV	699 (2)	−1^a^	−2^c^	0	−1^d^	0	⊕⊝⊝⊝ Very low quality
LVESV	699 (2)	−1^a^	−2^c^	0	−1^d^	0	⊕⊝⊝⊝ Very low quality
LVESD	162 (2)	−1^a^	0	0	−1^d^	0	⊕⊕⊝⊝ Low quality
Heartbeat recovery rate	238 (3)	−1^a^	−2^c^	0	−1^d^	0	⊕⊝⊝⊝ Very low quality
TCM chest pain symptom score	206 (3)	−1^a^	0	0	−1^d^	0	⊕⊕⊝⊝ Low quality
Duration of ICU stay	274 (5)	−1^a^	−2^c^	0	−1^d^	0	⊕⊝⊝⊝ Very low quality
Duration of hospital stay	320 (4)	−1^a^	−2^c^	0	−1^d^	0	⊕⊝⊝⊝ Very low quality
Incidence of MACE	492 (5)	−1^a^	0	0	0	0	⊕⊕⊕⊝ Moderate quality
6MWT	194 (3)	−1^a^	0	0	−1^d^	0	⊕⊕⊝⊝ Low quality
SAQ	192 (2)	−1^a^	0	0	−1^d^	0	⊕⊕⊝⊝ Low quality

^a^Method of assigning concealment is not described.

^b^75% > *I*^2^ > 50%.

^c^ *I* ^2^ ≥ 75%.

^d^Sample size < 400 or wide confidence intervals.

^e^Sample size < 400 with wide confidence intervals.

## Data Availability

All data in this study can be obtained through the corresponding author's email address, E-mail: acuresearch@126.com. All data in this study are available upon request from the corresponding author via email.

## Supporting Information

Supporting Figures 1–13: Forest plot of cTnI, CK-MB, BNP, hs-CRP, TNF-α, IL-10, IL-6, MDA, SOD, LVEF, LVEDD, LVEDV, and LVESV in acupuncture and control groups.
