# Correction to “A Meta‐Analysis of Randomized Controlled Trials (RCTs) Investigating the Efficacy and Safety of Acupuncture in Treating Myocardial Ischemia/Reperfusion (I/R) Injury”

**DOI:** 10.1155/crp/9872431

**Published:** 2026-03-01

J. Xiong, Y. Wei, X. Huang, et al., “A Meta‐Analysis of Randomized Controlled Trials (RCTs) Investigating the Efficacy and Safety of Acupuncture in Treating Myocardial Ischemia/Reperfusion (I/R) Injury,” *Cardiology Research and Practice*, no. 2025 (2025), https://doi.org/10.1155/crp/9970541.

In the article titled “A Meta‐Analysis of Randomized Controlled Trials (RCTs) Investigating the Efficacy and Safety of Acupuncture in Treating Myocardial Ischemia/Reperfusion (I/R) Injury,” there are errors in the legends of Figures 6, 9, and 17. These errors are corrected as follows:

1. Figure 6: “Forest plot of myocardial enzyme CK in acupuncture and control groups.” should read “Forest plot of myocardial enzyme LDH in acupuncture and control groups.”
2. Figure 9: “Forest plot of the inflammatory factor IL‐1 in acupuncture and control groups.” should read “Forest plot of the inflammatory factor IL‐8 in acupuncture and control groups.”
3. Figure 17: “Forest plot of MACE incidence in acupuncture and control groups.” should read “Forest plot of 6MWT incidence in acupuncture and control groups.”

The authors confirm that these errors do not affect the results and conclusions.

We apologize for these errors.

