# Supplementary figures and images for: A Meta-Analysis of Randomized Controlled Trials (RCTs) Investigating the Efficacy and Safety of Acupuncture in Treating Myocardial Ischemia/Reperfusion (I/R) Injury
Source: Cardiol Res Pract. 2025 Jun 24;2025:9970541. doi: 10.1155/crp/9970541 (PMC12213044; doi:10.1155/crp/9970541)

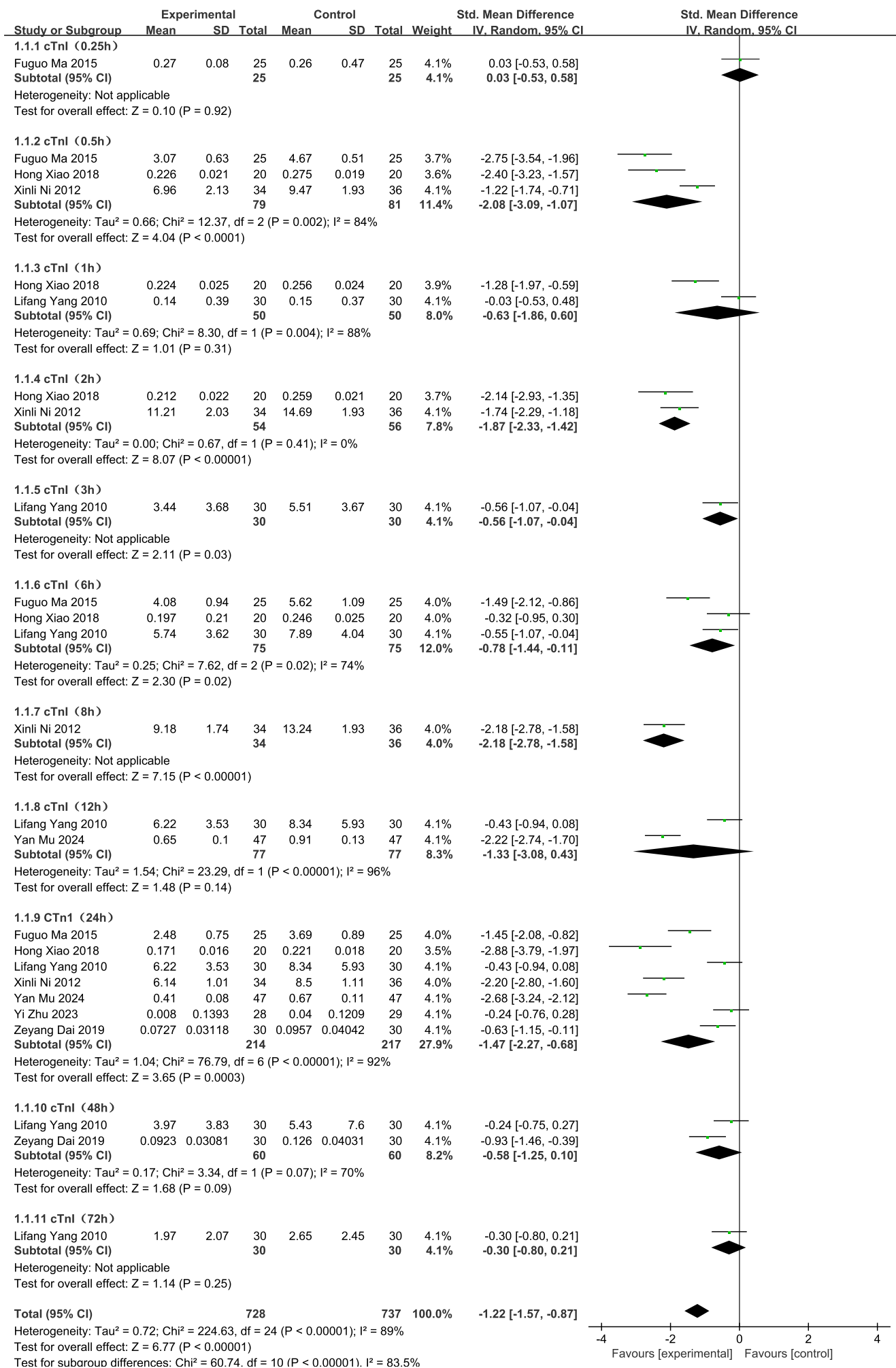

Supplement: Supporting Information — Additional supporting information can be found online in the Supporting Information section. [file 9970541.f1.zip › Supplementary Figure 1 for Forest map of cTnI.pdf]

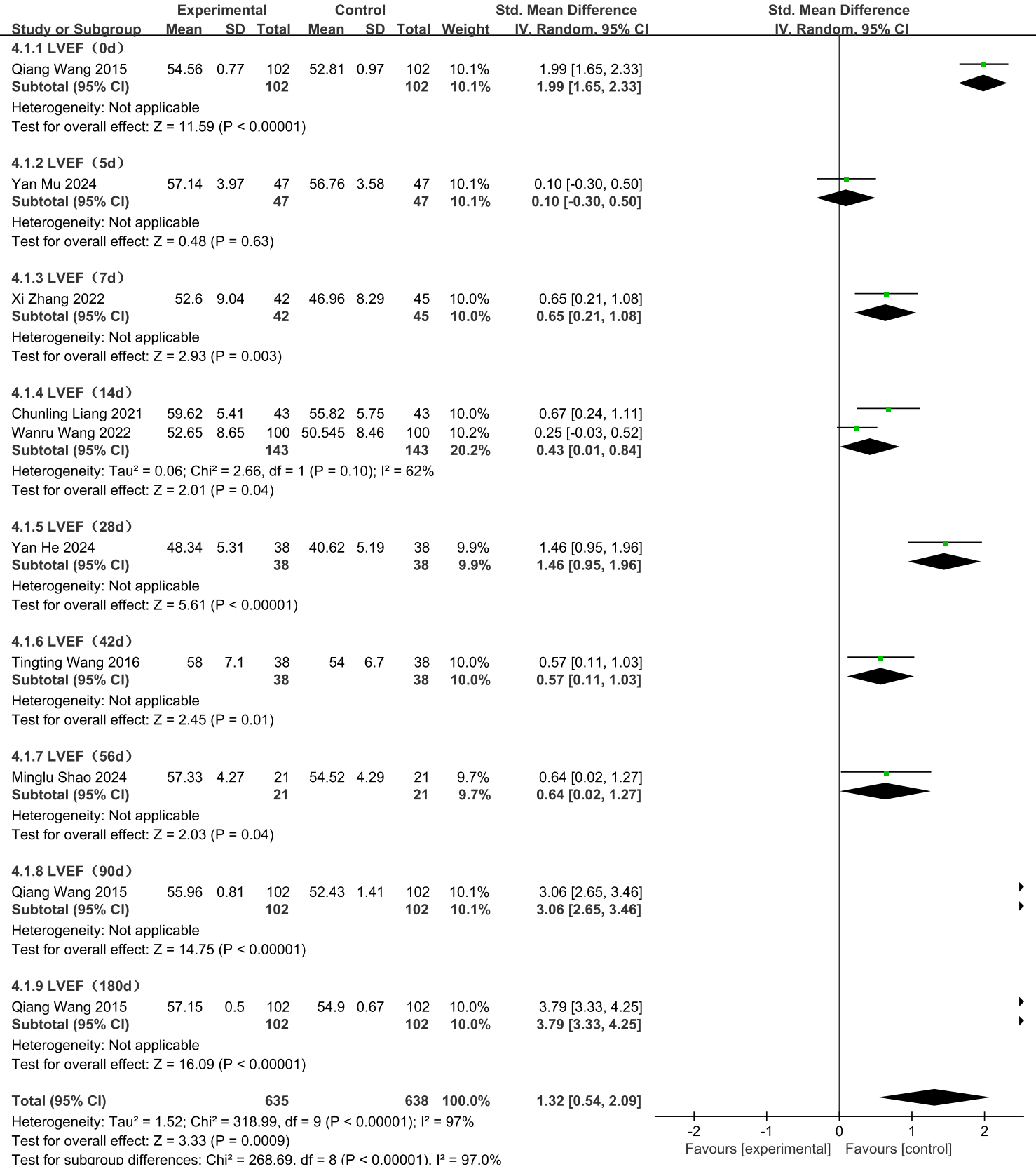

Supplement: Supporting Information — Additional supporting information can be found online in the Supporting Information section. [file 9970541.f1.zip › Supplementary Figure 10 for Forest map of LVEF.pdf]

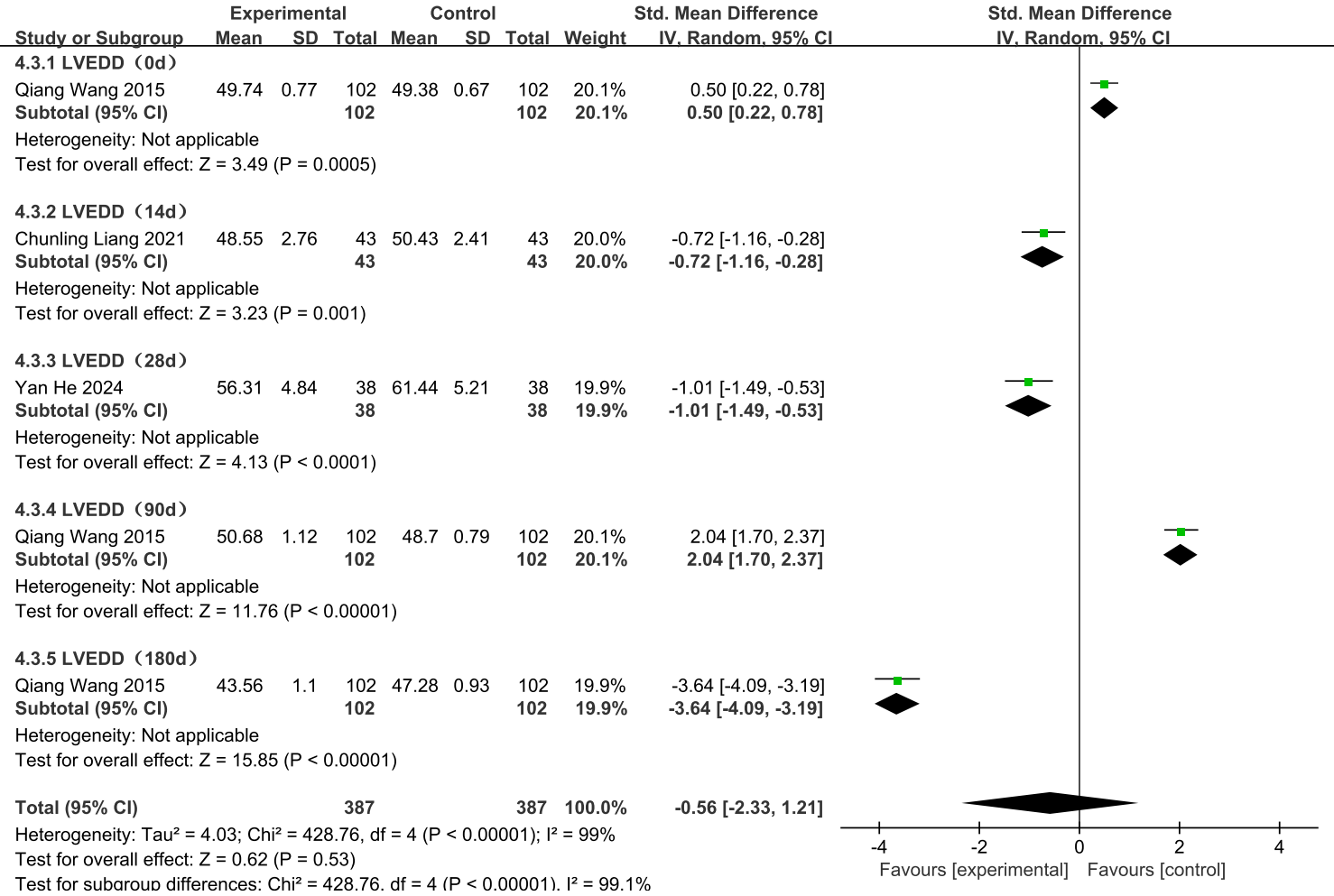

Supplement: Supporting Information — Additional supporting information can be found online in the Supporting Information section. [file 9970541.f1.zip › Supplementary Figure 11 for Forest map of LVEDD.pdf]

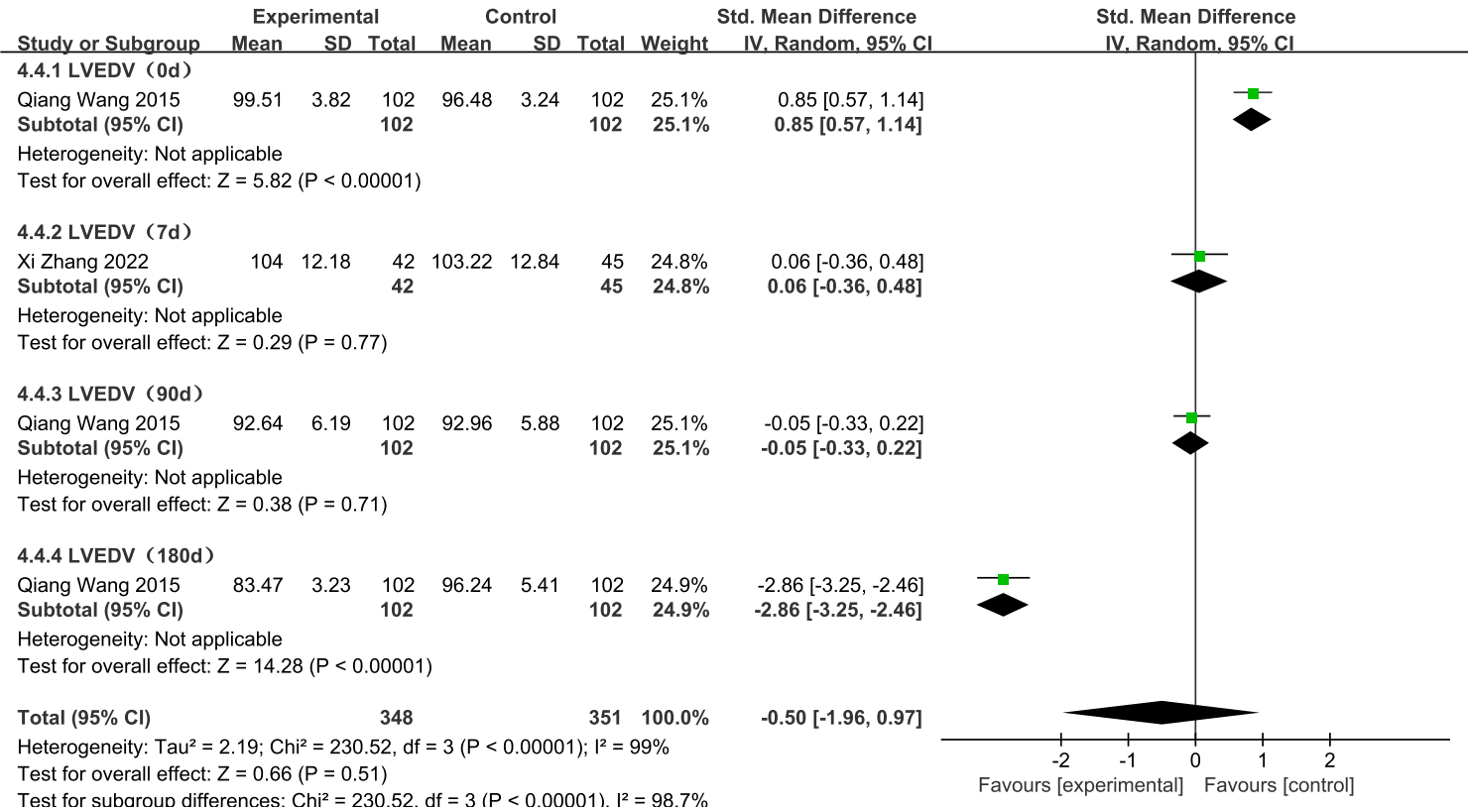

Supplement: Supporting Information — Additional supporting information can be found online in the Supporting Information section. [file 9970541.f1.zip › Supplementary Figure 12 for Forest map of LVEDV.pdf]

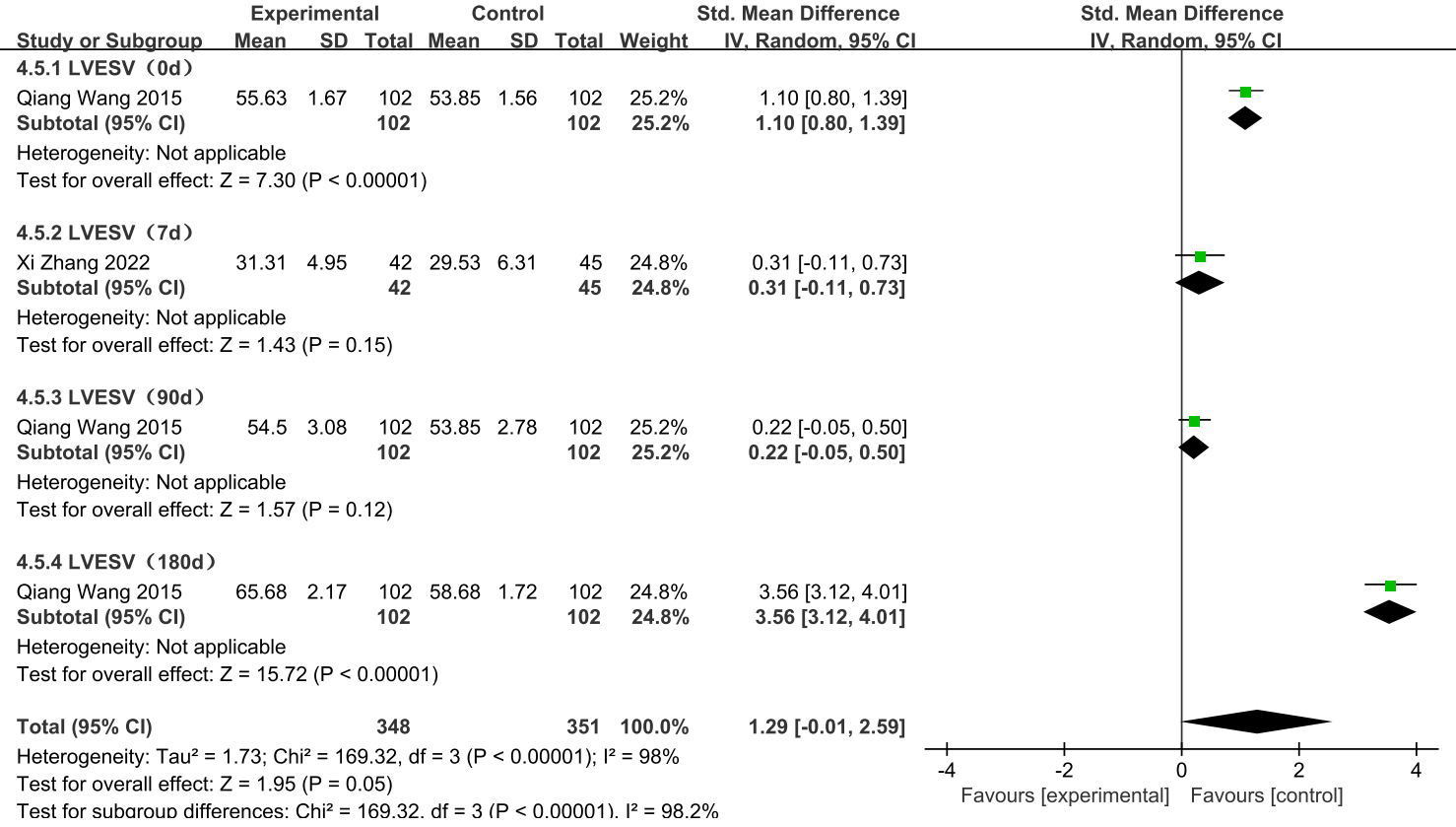

Supplement: Supporting Information — Additional supporting information can be found online in the Supporting Information section. [file 9970541.f1.zip › Supplementary Figure 13 for Forest map of LVESV.pdf]

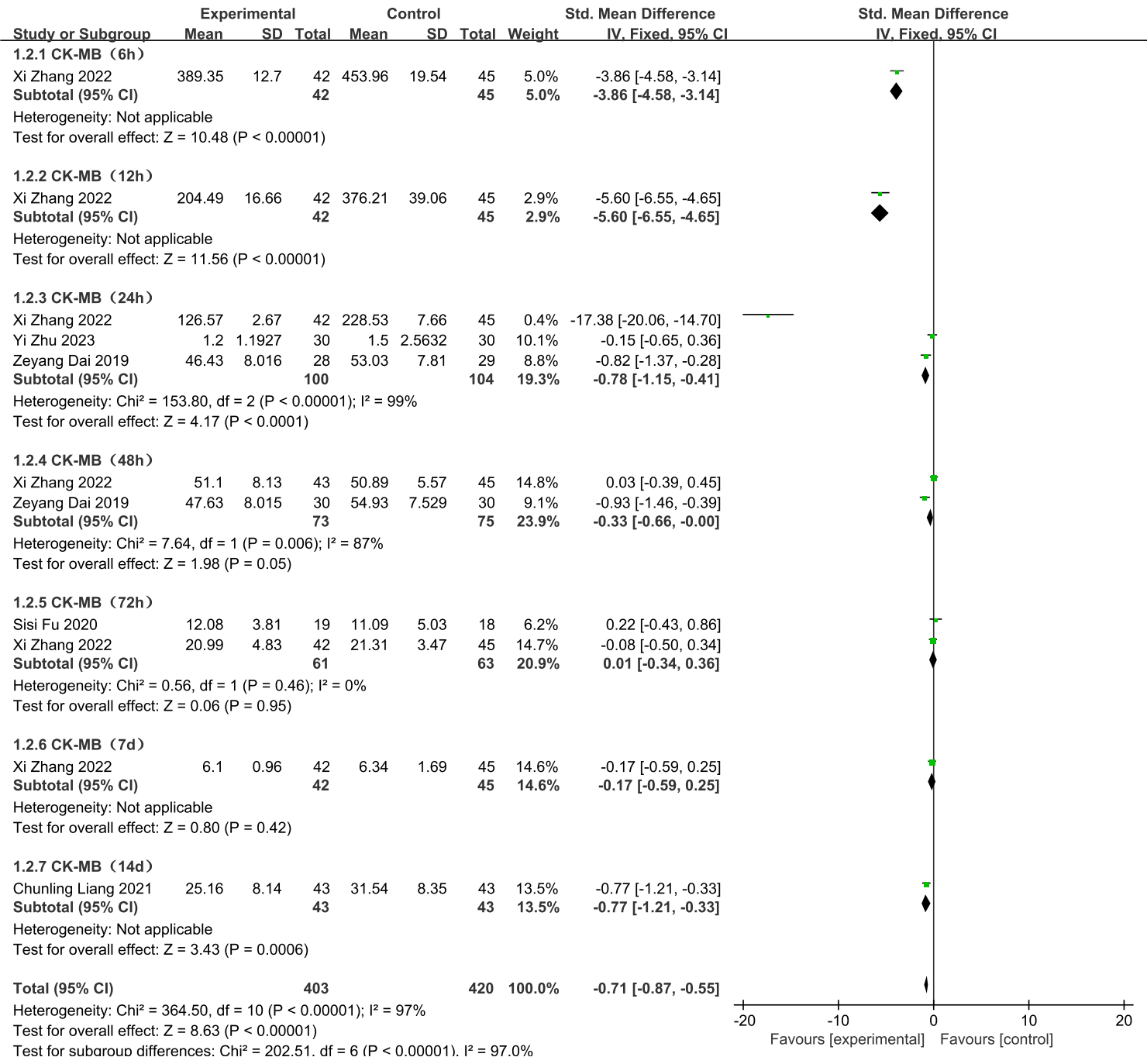

Supplement: Supporting Information — Additional supporting information can be found online in the Supporting Information section. [file 9970541.f1.zip › Supplementary Figure 2 for Forest map of CK-MB.pdf]

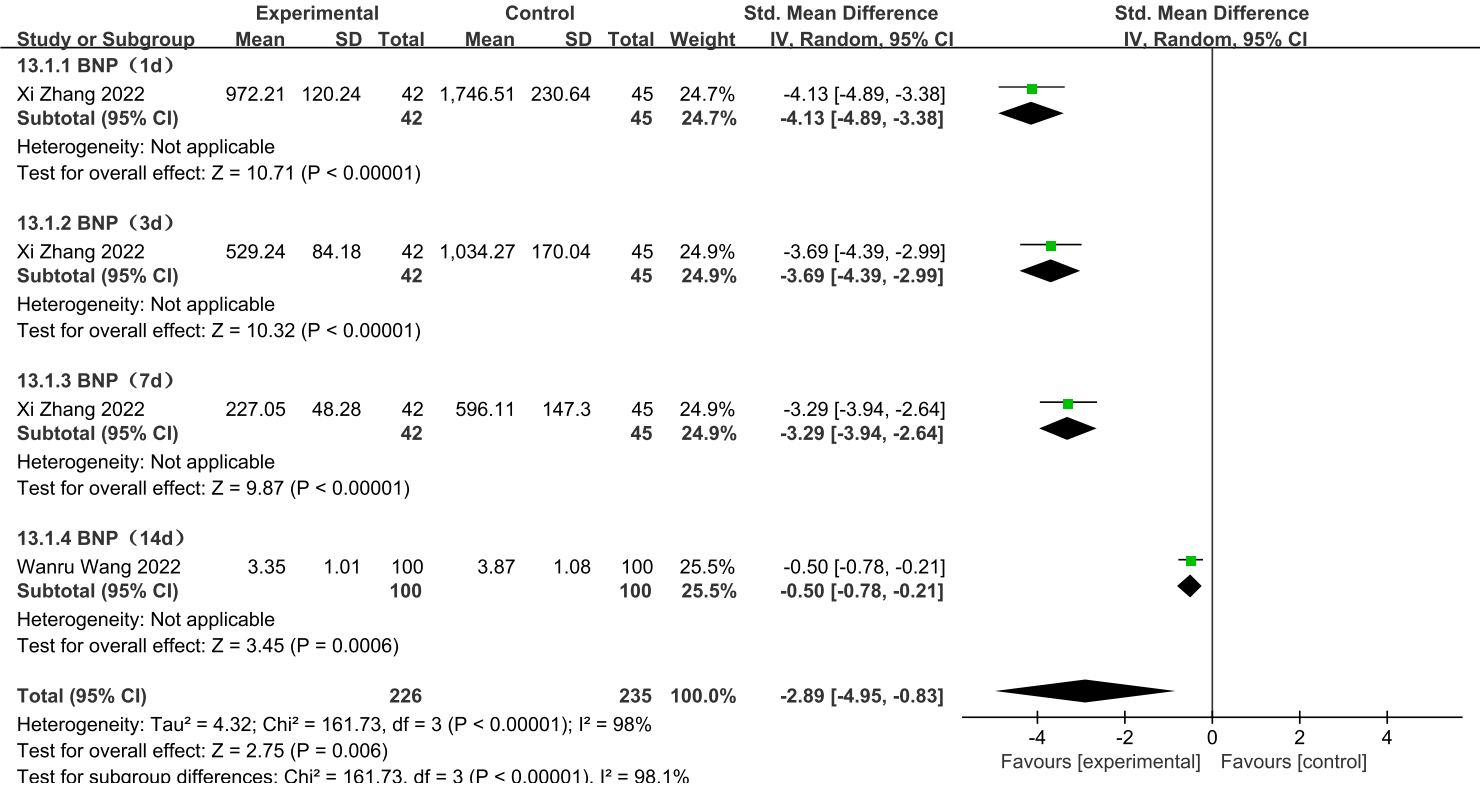

Supplement: Supporting Information — Additional supporting information can be found online in the Supporting Information section. [file 9970541.f1.zip › Supplementary Figure 3 for Forest map of BNP.pdf]

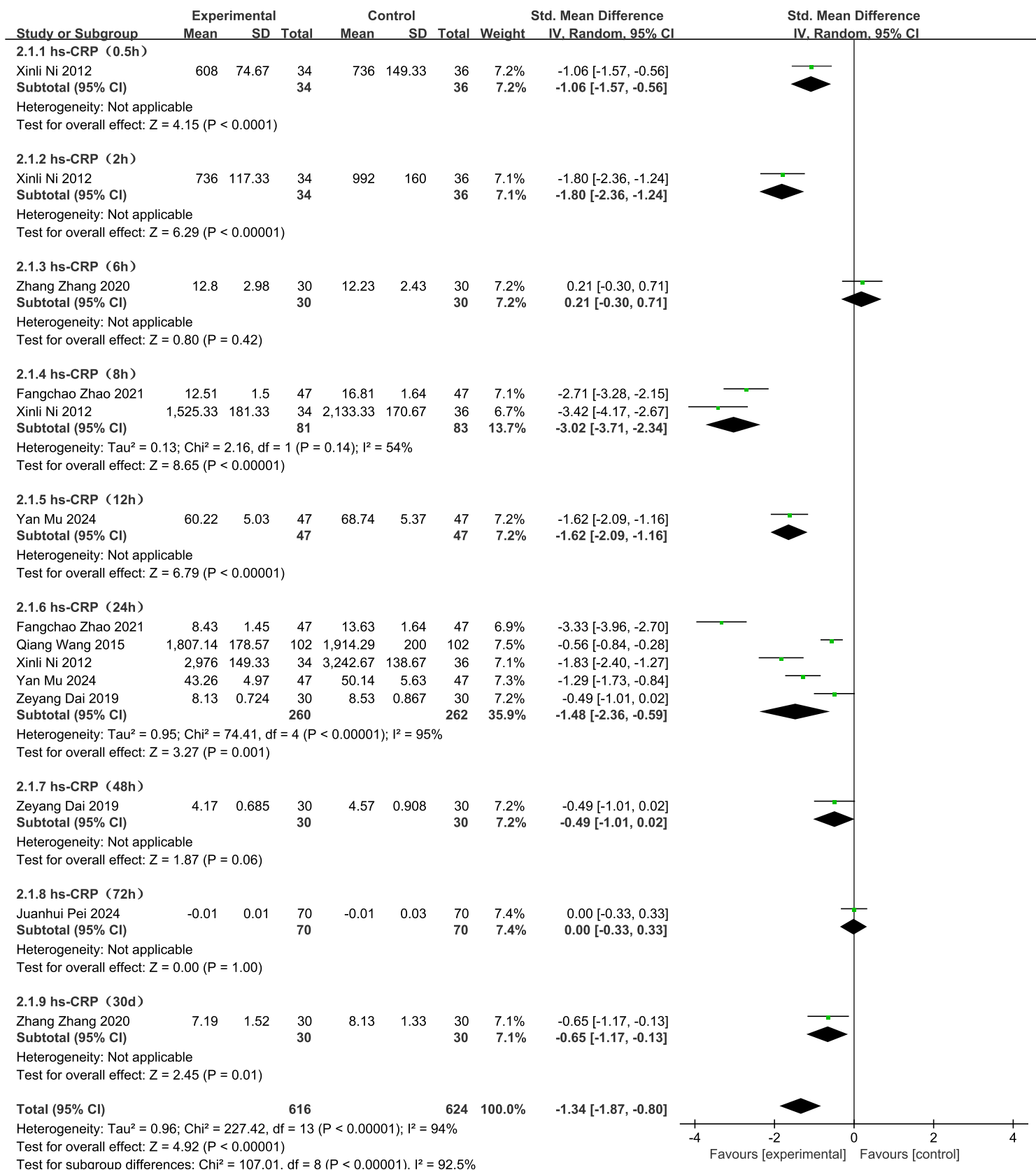

Supplement: Supporting Information — Additional supporting information can be found online in the Supporting Information section. [file 9970541.f1.zip › Supplementary Figure 4 for Forest map of hs-CRP.pdf]

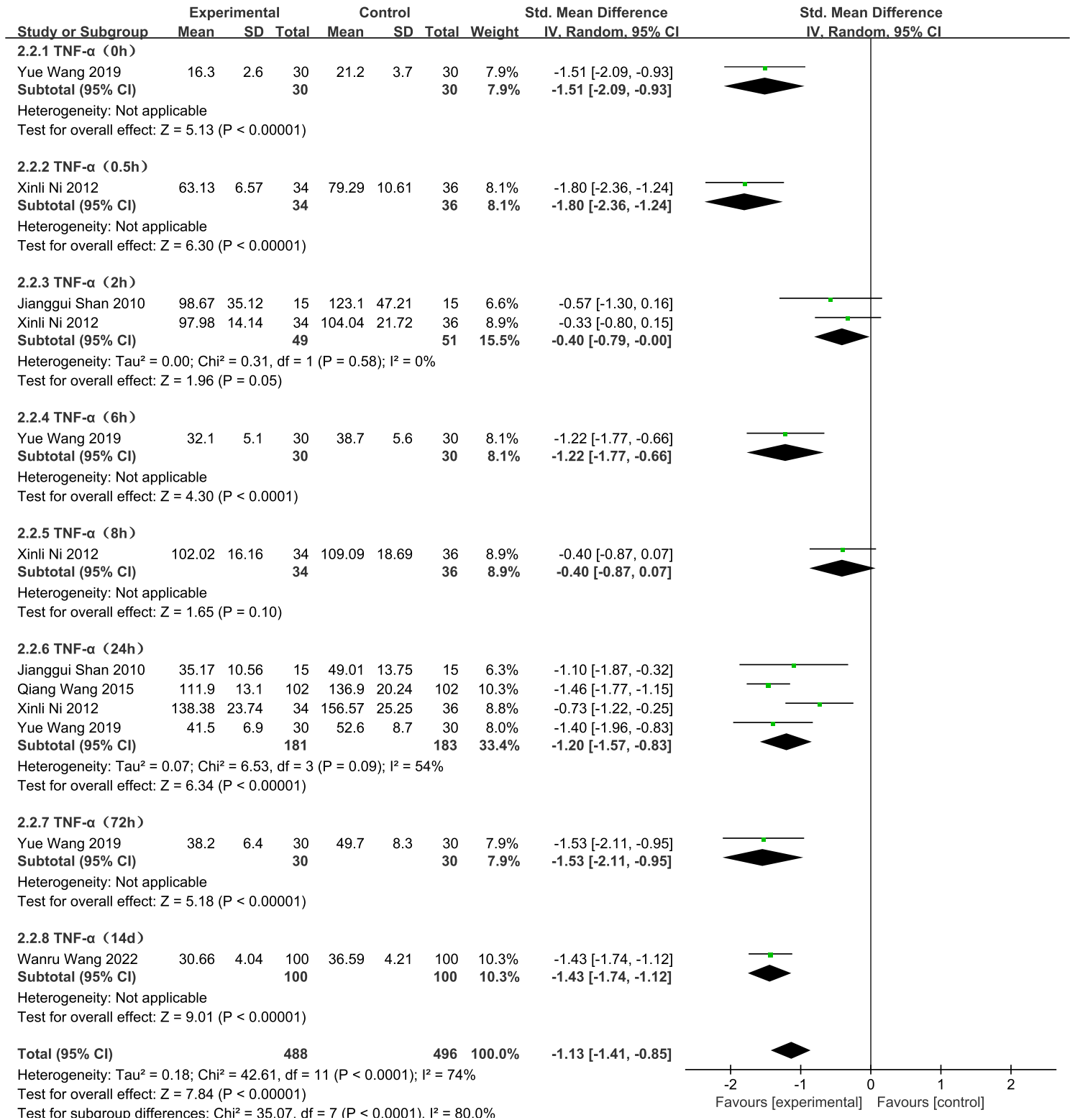

Supplement: Supporting Information — Additional supporting information can be found online in the Supporting Information section. [file 9970541.f1.zip › Supplementary Figure 5 for Forest map of TNF-α.pdf]

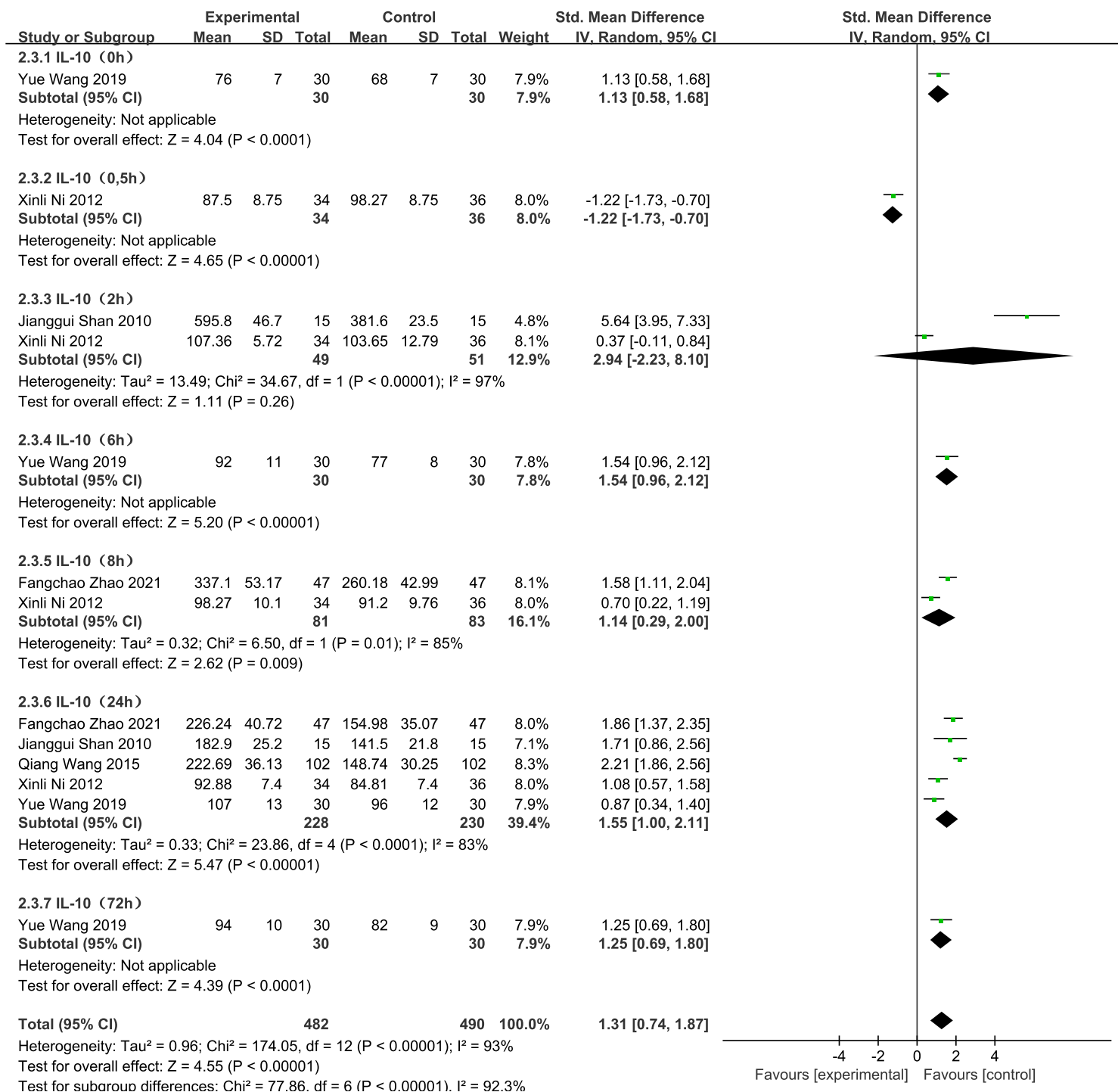

Supplement: Supporting Information — Additional supporting information can be found online in the Supporting Information section. [file 9970541.f1.zip › Supplementary Figure 6 for Forest map of of IL-10.pdf]

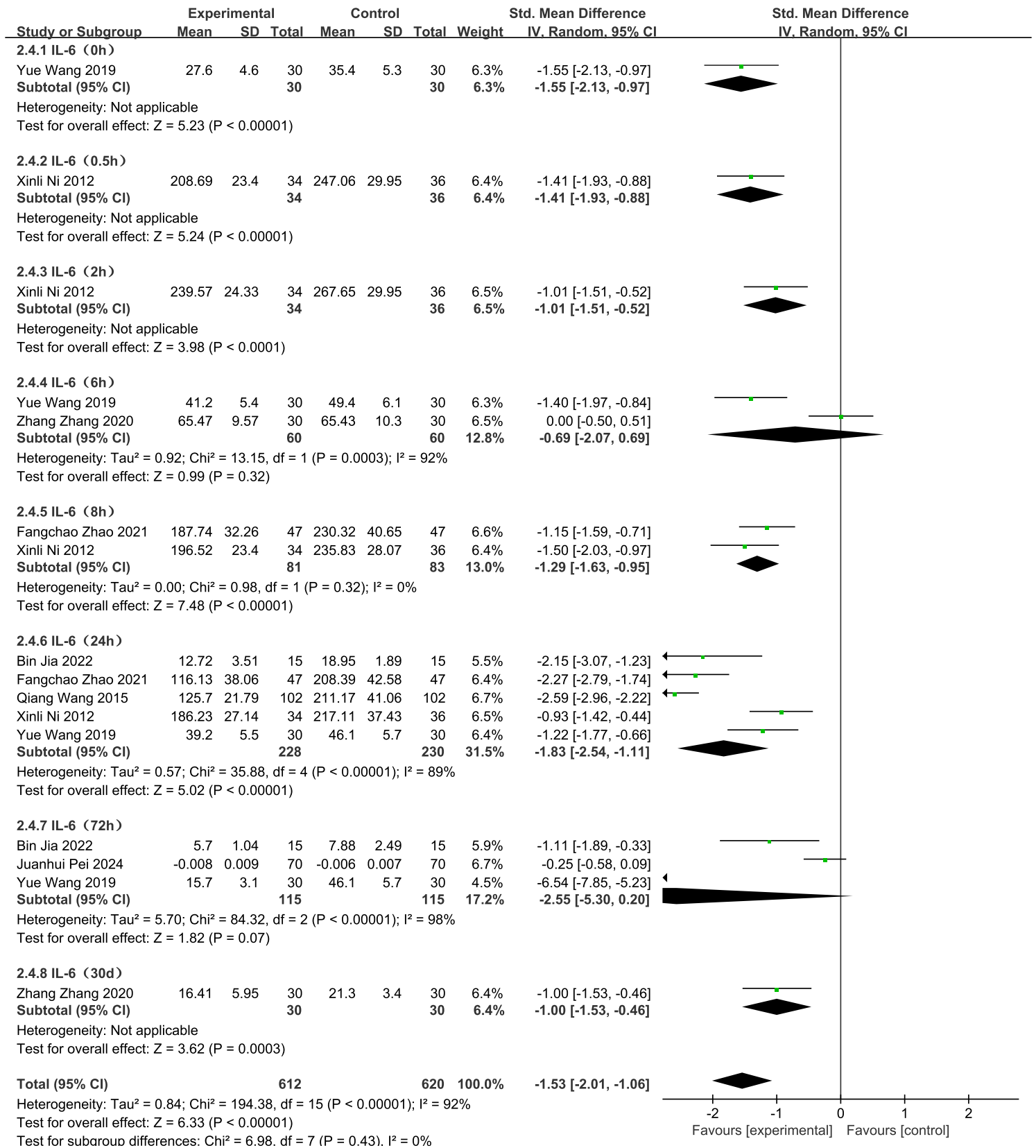

Supplement: Supporting Information — Additional supporting information can be found online in the Supporting Information section. [file 9970541.f1.zip › Supplementary Figure 7 for Forest map of of IL-6.pdf]

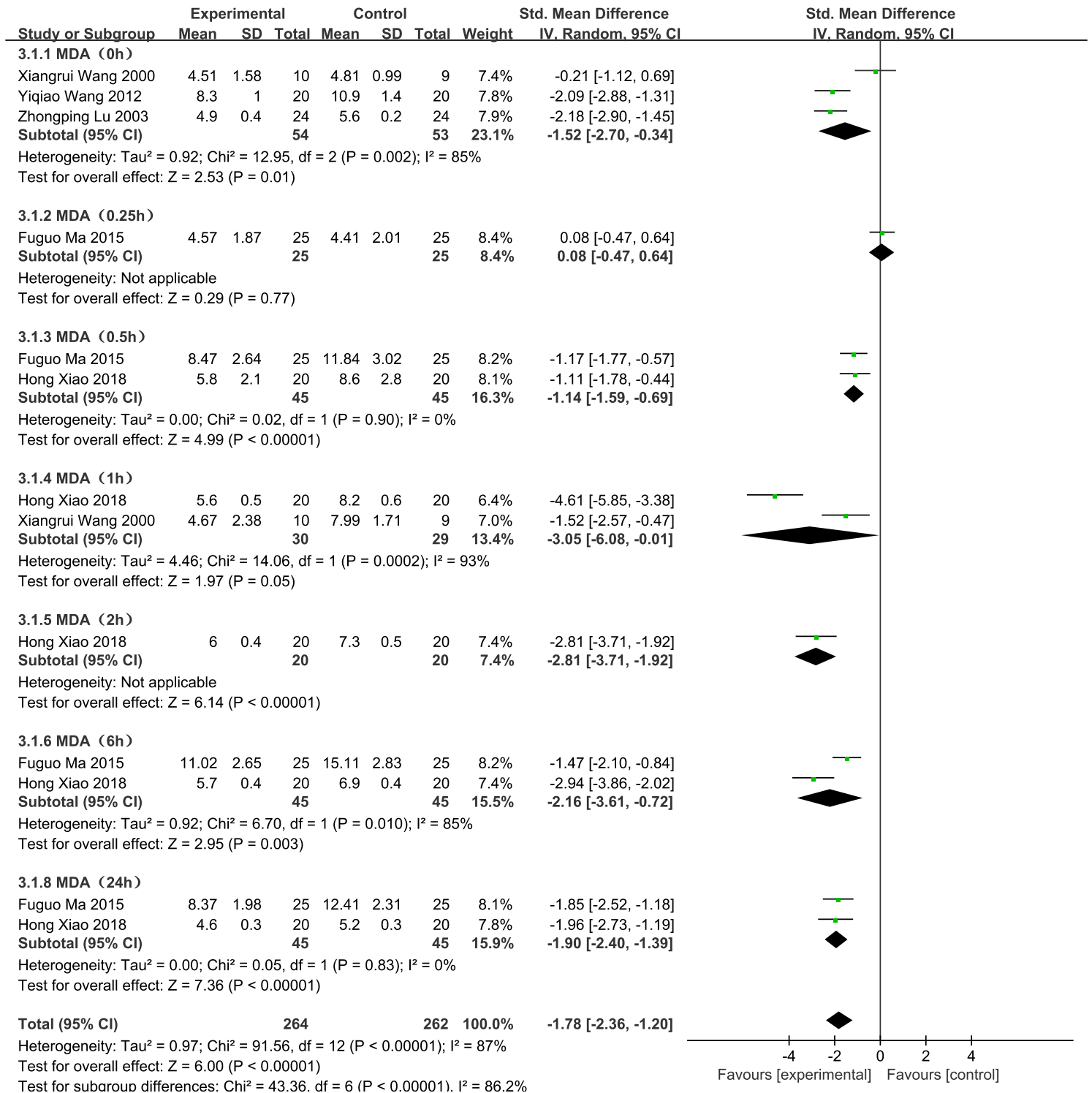

Supplement: Supporting Information — Additional supporting information can be found online in the Supporting Information section. [file 9970541.f1.zip › Supplementary Figure 8 for Forest map of MDA.pdf]

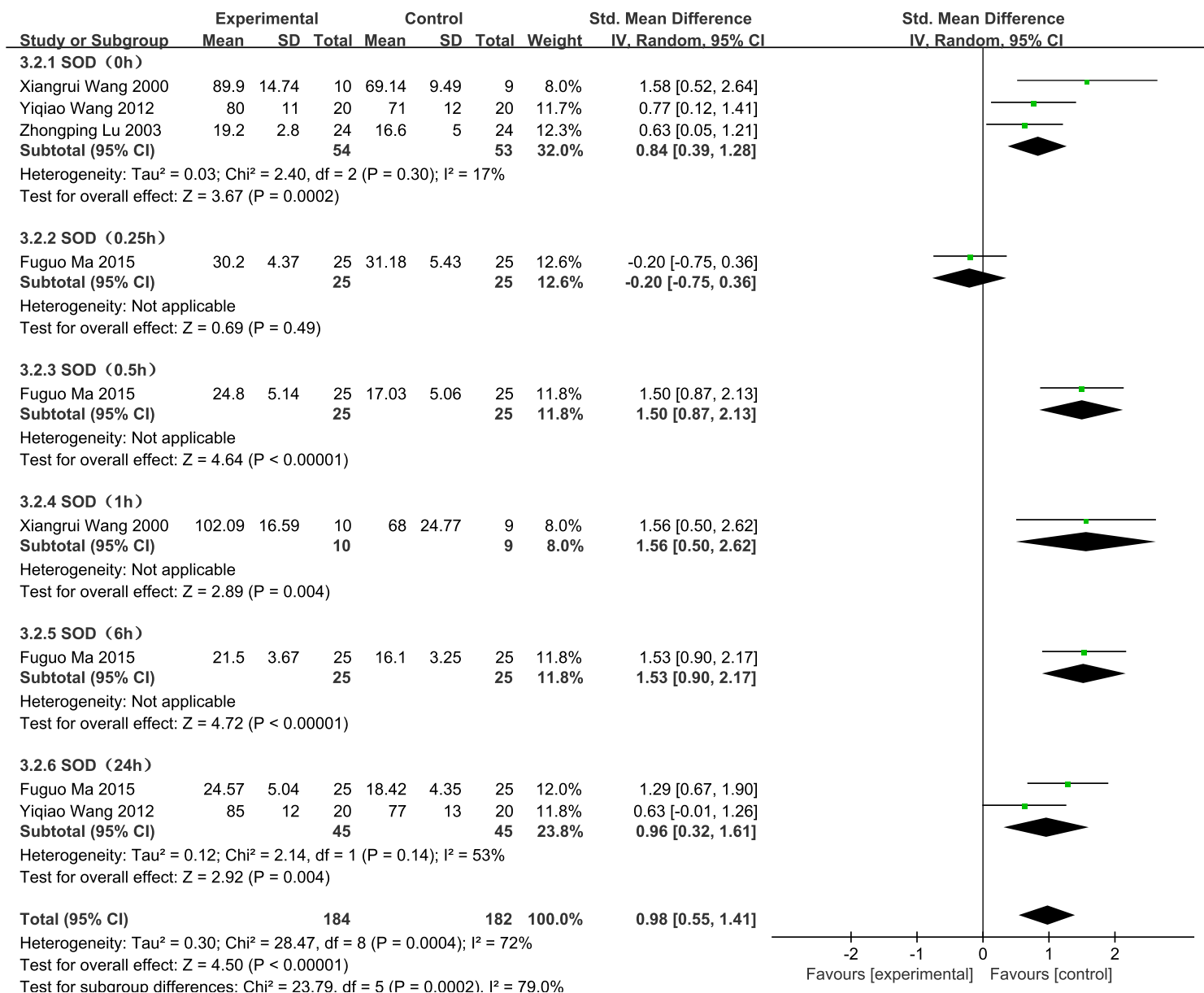

Supplement: Supporting Information — Additional supporting information can be found online in the Supporting Information section. [file 9970541.f1.zip › Supplementary Figure 9 for Forest map of SOD.pdf]
